# Mechanisms and regulation of defensins in host defense

**DOI:** 10.1038/s41392-023-01553-x

**Published:** 2023-08-14

**Authors:** Jie Fu, Xin Zong, Mingliang Jin, Junxia Min, Fudi Wang, Yizhen Wang

**Affiliations:** 1 Key Laboratory of Molecular Animal Nutrition, Ministry of Education, College of Animal Sciences, Zhejiang University, Hangzhou, China; 2 Key Laboratory of Animal Nutrition and Feed Science in Eastern China, Ministry of Agriculture, Hangzhou, Zhejiang Province China; 3 The First Affiliated Hospital, Institute of Translational Medicine, Zhejiang University School of Medicine, Hangzhou, 310058 China; 4 The Second Affiliated Hospital, School of Public Health, State Key Laboratory of Experimental Hematology, Zhejiang University School of Medicine, Hangzhou, China; 5 The First Affiliated Hospital, Basic Medical Sciences, School of Public Health, Hengyang Medical School, University of South China, Hengyang, China

**Keywords:** Immunology, Molecular biology, Diseases

## Abstract

As a family of cationic host defense peptides, defensins are mainly synthesized by Paneth cells, neutrophils, and epithelial cells, contributing to host defense. Their biological functions in innate immunity, as well as their structure and activity relationships, along with their mechanisms of action and therapeutic potential, have been of great interest in recent years. To highlight the key research into the role of defensins in human and animal health, we first describe their research history, structural features, evolution, and antimicrobial mechanisms. Next, we cover the role of defensins in immune homeostasis, chemotaxis, mucosal barrier function, gut microbiota regulation, intestinal development and regulation of cell death. Further, we discuss their clinical relevance and therapeutic potential in various diseases, including infectious disease, inflammatory bowel disease, diabetes and obesity, chronic inflammatory lung disease, periodontitis and cancer. Finally, we summarize the current knowledge regarding the nutrient-dependent regulation of defensins, including fatty acids, amino acids, microelements, plant extracts, and probiotics, while considering the clinical application of such regulation. Together, the review summarizes the various biological functions, mechanism of actions and potential clinical significance of defensins, along with the challenges in developing defensins-based therapy, thus providing crucial insights into their biology and potential clinical utility.

## Introduction

Host defense peptides (HDPs) are polypeptides assembled from fewer than 100 amino acids. These peptides tend to have a high proportion of positively charged and hydrophobic residues.^1,2^ Based on the Host Defense Peptides Database in 2022 (https://wangapd3.com/main.php), scientists have identified or predicted a total of 3257 HDPs. These HDPs are derived from various organisms, including 365 from bacteria, five from archaea, eight from protists, 30 from fungi, 371 from plants, and 2521 from animals. Among these, 348 are classified as defensins and have an average length of 41.26 residues, with an average net charge of 4.61.

Based on the amino acid composition, length and structural characteristics, mammalian HDPs are generally categorized into two prominent families: defensins and cathelicidins. The cathelicidins comprise a conserved gene family, initially thought to produce small proteins with cysteine protease inhibitor activity, as well as antimicrobial activity.^3,4^ Recently, however, the notion of protease inhibitor activity of cathelicidins has been refuted.^5^ Although pro-defensins are inactive, pro-cathelicidins and cathelicidins are equally bactericidal.^5^ Initially, direct activity against microorganisms was deemed to be the primary role of HDPs. For example, mouse cryptdins and human alpha defensin-5 (HD5) directly kill *Salmonella*, and human alpha defensin 6 (HD6) traps *Salmonella* in a high-ordered “nanonet” structure to prevent infection.^6,7^ However, many HDPs lose their antimicrobial potency in some localized microenvironments. Even so, it is becoming increasingly clear that HDPs act as immunomodulatory mediators that regulate the mammalian innate immune response and moderate the establishment of adaptive immunity.^8,9^ The structure, function and mechanism of action of cathelicidins^4,10–13^ and defensins^14–20^ have been reviewed over the past few years. Nonetheless, given the enormous number of defensins known, the diversity of their biological activities, the intricate ways in which they function, and the multitude of targets they interact with, publishing a comprehensive review on this topic is an arduous, if not impossible, feat.

Thus, this review is focused on defensins in host defense. It mainly summarizes and discusses their properties, biological function, related clinical diseases, and therapeutic potential, as well as their nutritional regulation. We will also cover the function of defensins in promoting the chemotaxis of immune cells, their influence on multiple signaling pathways involved in inflammation and immunity, how they maintain gut microbial homeostasis and their regulation of epithelial injury and the promotion of proper organ development and eukaryotic cell death, as well as their contribution to clinical diseases and their therapeutic potential. We will highlight the current knowledge base regarding mammalian defensins and their roles in regulating host health, thus providing a theoretical basis for clinical therapeutic strategies targeting defensins to treat disease.

## History of defensins

**Fig. 1:**
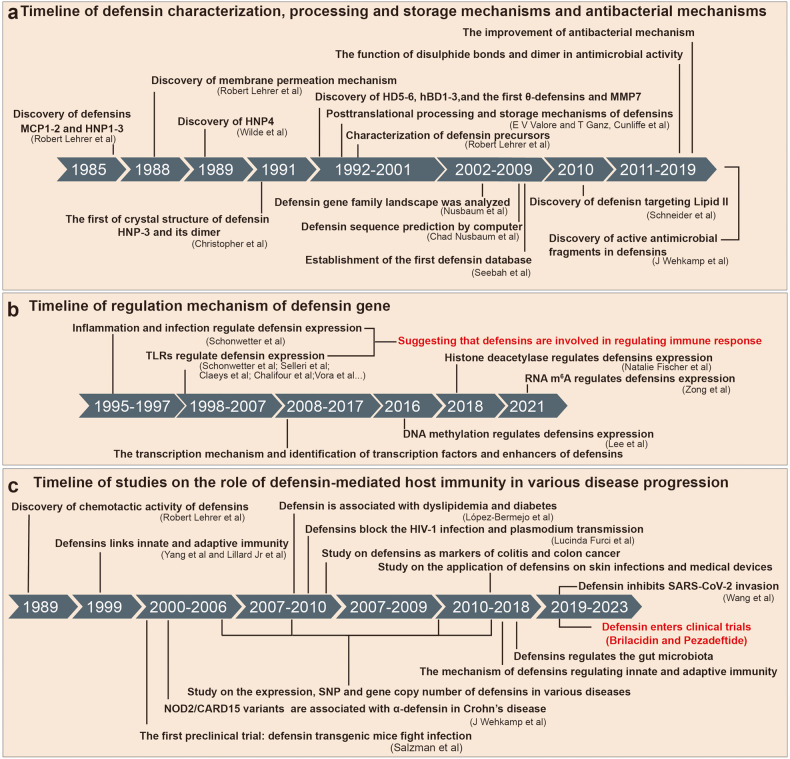
Introduction to the history of defensin research. **a** Timeline of defensin characterization, processing and storage mechanisms and antibacterial mechanisms. **b** Timeline of regulation mechanism of defensin gene. **c** Timeline of studies on the role of defensin-mediated host immunity in various disease progression. SNP single-nucleotide polymorphism

In 1985, Dr. Robert Lehrer from the University of California, Los Angeles, was the first to discover and name defensins. He reported that rabbit defensins MCP-1 and MCP-2 had strong antibacterial and antiviral activities^21,22^ (Fig. 1a). That same year, he and his team discovered and characterized the structure of human neutrophil peptides (HNP1-3)^23^ (Fig. 1a). Over time, more defensins were found, such as HNP4 (ref. ^24^) in 1989, HD5 (ref. ^25^) and HD6 (ref. ^26^) in 1992 and 1993, respectively, and human β-defensins (hBD1-3)^27–29^ in 1995, 1997, and 2001, respectively (Fig. 1a). The first θ-defensin was found in 1999 (ref. ^30^) (Fig. 1a). Since then, with the widespread use of in silico analyses, researchers have been able to predict the sequence and structure of defensins^31^ (Fig. 1a). Meanwhile, in the late 20th and early 21th centuries, the scientific community widely studied the processing and storage mechanisms of defensins^6,32–36^ (Fig. 1a). In addition, from 1988 to 2010, the antibacterial mechanisms of defensins have been established, which involve a membrane penetration mechanism and targeting lipid II by inhibiting cell wall synthesis^37,38^ (Fig. 1a). During this period, the role of defensin dimers, disulfide bonds and other biochemical structures in their antibacterial function and mechanisms have also been analyzed^39–43^ (Fig. 1a).

In 2007, chromosome 8 was fully sequenced and analyzed by the Human Genome Project, resulting in the description of the first human defensin gene family landscape^44–46^ (Fig. 1a). The first defensin database was established in the same year, incorporating 350 defensins^47^ (Fig. 1a). Then, in the early decade of the 21st century, researchers gradually analyzed the regulation pattern of defensin gene expression^48–53^ (Fig. 1b). These results provide essential data and technical support for the subsequent research into the genetic engineering and drug development of defensins.

Over the last two decades, defensins have been found to regulate immune cell chemotaxis and to be involved in regulating sperm activity, male infertility, thrombosis, melanin deposition, and other essential biological functions.^54–58^ Further, defensins have also been shown to induce the host’s innate immune response, enhance the host’s adaptive immune response and promote the activation of T cells, macrophages, and other immune cells^59–65^ (Fig. 1c). Importantly, the role and mechanism of defensins in regulating immune responses have been fully analyzed. Since the 2010s, defensins have been used in the biomedical field.^66^ For example, they have been applied to the surface of medical instruments to produce long-lasting and broad-spectrum antibacterial activity^67,68^ (Fig. 1c). Also, with the development of gene editing and peptide chemical synthesis technologies, many preclinical studies have been conducted on a variety of diseases and tumors via the use of transgenic mouse models of defensins and oral or injected recombinant defensins, and their regulatory role and precise mechanisms in different diseases and tumors have been explored in depth^69–79^ (Fig. 1c). Thus, the interaction network of defensins regulating host immune homeostasis has been constructed, and several reliable drug targets have been identified. In recent years, two defensins have entered clinical trials (Fig. 1c). In conclusion, with the continuous development of science and technology, the study of defensins is deepening, becoming an essential tool and resource to achieve biological protection and human health.

## Structural features and evolution

**Fig. 2:**
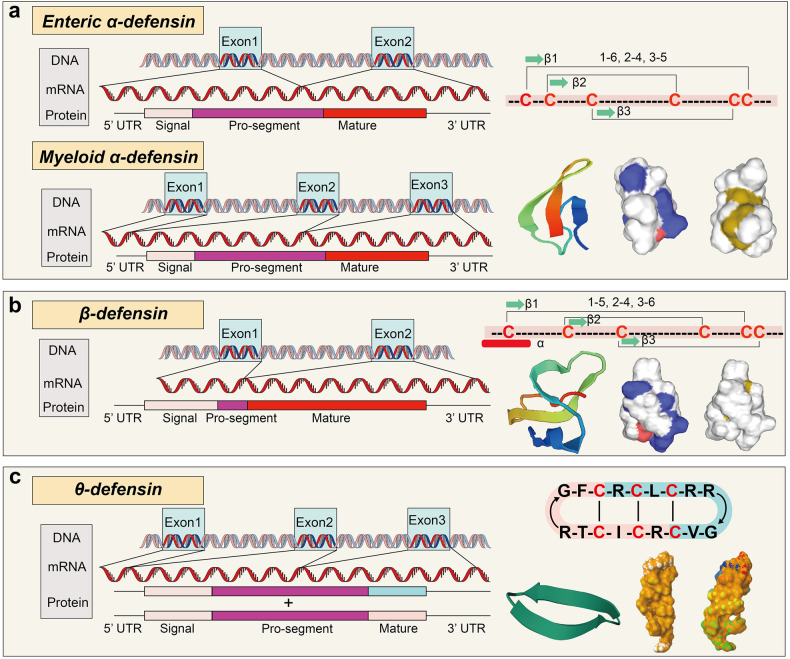
Structural characteristics of defensins from gene to mRNA to protein. The structure of defensin genes and peptides, including the alignment of the enteric and myeloid α-defensins (**a**, UniProt: P59665), β-defensin (**b**, UniProt: P60022), and θ-defensin (**c**, UniProt: P82271) genes are indicated, along with the number of exons and the coding of signal peptides, pro-segment and mature peptides, as well as the location and the disulfide pairing of cysteines and the helical wheel plots and three-dimensional structure

Most defensins are cationic peptides of 18–45 amino acids. They have six conserved cysteines that allow for three intramolecular disulfide bonds that stabilize the peptide.^80^ The essential information and structural characteristics of most human, mouse, pig, and bovine defensins are listed in Table 1. Mammalian defensins are categorized as α-, β-, and θ-defensins, based on amino acid homology and cysteine residue connectivity^19^ (Fig. 2). However, humans only produce α- and β-defensins.^81,82^ Despite having differing covalent structures, α- and β-defensins have similar tertiary structures (Fig. 2 and Table 1). The gene clusters that encode the α-defensin subfamily and most β-defensin subfamily are situated on chromosome 8 (ref. ^83^) (Table 1), with α-defensin genes deriving from β-defensin genes.^84,85^ In addition, mammalian defensin genes evolved rapidly, and some newly discovered hBDs are encoded by genes on chromosomes 11 and 20 (Table 1). Through in situ hybridization studies, it has been revealed that the defensin genes clustered on chromosome 20 are transcribed at different locations in the epididymis,^86^ and there is evidence that they are involved in sperm chemotaxis and maturation and associated with idiopathic infertility.^19,56,87–89^

**Table 1:** Various characteristic features of human, mouse, pig, and bovine defensins

Defensin name	Abbreviation	Chromosome	UniProt ID	Signal peptide	Mature defensin	Disulfide pattern in mature defensin
Human α-defensin						
Neutrophil defensin-1	HNP1	8	P59665	1–19	66–94	66–94, 68–83, 73–93
Neutrophil defensin 2	HNP2	8	P59666	1–19	64–94	66–94, 68–83, 73–93
Neutrophil defensin 3	HNP3	8	P59666	1–19	65–94	66–94, 68–83, 73–93
Neutrophil defensin 4	HNP4	8	P12838	1–19	66–96	65–93, 67–82, 72–92
Human α-defensin-5	HD5	8	Q01523	1–19	63–94	65–93, 67–82, 72–92
Human α-defensin 6	HD6	8	Q01524	1–19	69–100	72–99, 74–88, 78–98
Human β-defensin						
Human β-defensin-1	hBD1	8	P60022	1–21	33–68	37–66, 44–59, 49–57
Human β-defensin 2	hBD2	8	O15263	1–23	24–64	31–60, 38–53, 43–61
Human β-defensin 103A	hBD3	8	P81534	1–22	23–67	33–62, 40–55, 45–63
Human β-defensin 104A	hBD4	8	Q8WTQ1	1–22	23–72	30–57, 37–51, 41–58
Human β-defensin 105A	hBD5	8	Q8IZN8	1–27	28–78	43–74, 53–67, 57–73
Human β-defensin 6	hBD6	8	Q496I8	1–20	21–65	26–53, 33–47, 37–54
Human β-defensin 109B	hBD9	8	Q30KR1	1–22	23–87	31–59, 38–53, 43–60
Human β-defensin 127	hBD27	20	Q9H1M4	1–20	21–63	24–53, 33–47, 37–54
Human β-defensin 119	DEFB20	20	Q8N690	1–21	22–84	28–55, 35–49, 39–56
Human β-defensin 126	hBD26	20	Q9BYW3	1–20	21–111	27–58, 34–52, 38–59
Human β-defensin 118	DEFB18	20	Q8N690	1–19	20–62	27–54, 34–48, 38–55
Human β-defensin 132	hBD32	20	Q7Z7B7	1–22	23–95	27–55, 35–49, 39–56
Human β-defensin 107A	hBD7	8	Q8IZN7	1–20	21–65	26–53, 33–47, 37–54
Human β-defensin 114	DEFB14	6	Q30KQ6	1–26	27–69	29–57, 36–60, 40–58
Human β-defensin 108B	hBD8	11	Q496I8	1–26	27–56	27–55, 35–39, 37–56
Mouse α-defensin						
Mouse α-defensins-1	Cryp1	8	P11477	1–19	59–93	64–92, 66–81, 71–91
Mouse α-defensins 2	Cryp2	8	Q8C1N9	1–19	20–95	
Mouse α-defensins 3	Cryp3	8	P28310	1–16	59–93	64–92, 66–81, 71–91
Mouse α-defensins 4	Cryp4	8	P28311	1–19	59–92	64–89, 66–81, 71–88
Mouse α-defensins 5	Cryp5	8	L7N230	1–19	20–93	
Mouse α-defensins 6	Cryp6	8	P28310	1–19	61–93	64–92, 66–81, 71–91
Mouse α–defensins 17	Cryp17	8	P28310	1–16	59–93	64–92, 66–81, 71–91
Mouse β-defensin						
Mouse β-defensin-1	mBD1	8	P56386	1–21	33–69	37–66, 44–59, 49–67
Mouse β-defensin 2	mBD2	8	P82020	1–20	21–71	37–66, 44–59, 49–67
Mouse β-defensin 3	mBD3	8	Q9WTL0	1–20	23–63	31–59, 38–52, 42–60
Mouse β-defensin 4	mBD4	8	P82019	1–22	23–63	31–59, 38–52, 42–60
Mouse β-defensin 6	mBD6	8	Q91VD6	1–22	23–63	31–59, 38–52, 42–60
Mouse β-defensin 7	mBD7	8	Q91V70	1–22	26–71	31–58, 38–52, 42–59
Mouse β-defensin 9	mBD9	8	Q8R2I6	1–24	25–67	34–62, 41–55, 45–63
Mouse β-defensin 10	mBD10	8	Q8R2I8	1–23	24–73	37–66, 44–59, 49–67
Mouse β-defensin 11	mBD11	8	Q8R2I7	1–23	24–77	37–66, 44–59, 49–67
Mouse β-defensin 12	mBD12	8	Q8K4N3	1–27	28–78	46–73, 53–67, 57–74
Mouse β-defensin 14	mBD14	8	Q7TNV9	1–22	23–67	33–62, 40–55, 45–63
Mouse β-defensin 19	mBD19	8	Q8K3I8	1–19	20–83	27–54, 34–48, 38–55
Mouse β-defensin 20	mBD20	2	Q30KP3	1–21	22–96	24–52, 32–46, 36–53
Mouse β-defensin 29	mBD29	2	Q8BGW9	1–23	24–78	40–67, 47–61, 51–68
Mouse β-defensin 30	mBD30	14	Q30KN4	1–22	23–75	35–62, 42–56, 46–63
Mouse β-defensin 41	mBD17	8	Q8K3I8	1–19	20–65	35–63, 42–56, 46–64
Mouse β-defensin 42	mBD42	14	Q8BVB5	1–21	22–75	33–60, 40–54, 44–61
Pig β-defensin						
Pig β-defensin-1	pBD1	15	O62697	1–20	24–64	31–60, 38–53, 43–61
Pig β-defensin 2	pBD2	15	Q6R953	1–21	22–69	37–65, 44–59,49–66
Pig β-defensin 128	pBD128	17	A0A287BN95	1–19	20–94	25–52, 32–46, 36–53
Pig β-defensin 121	pBD123	17	A0A8E8LS78	1–19	20–60	22–49, 29–43, 33–50
Pig β-defensin 110	pBD110	7	A0A287BBL9	1–19	20–67	35–63, 42–56, 46–64
Bovine β-defensin						
Bovine β-defensin 4	BNBD4	27	P46162	1–22	23–63	31–60, 38–53, 43–61
Bovine β-defensin-5	BNBD5	27	P46163	1–22	23–64	31–60, 38–53, 43–61
Bovine β-defensin 7	BNBD7	27	P46165	1–22	23–62	31–60, 38–53, 43–61
Bovine β-defensin 10	BNBD10	27	P46168	1–22	23–62	31–60, 38–53, 43–61
Bovine β-defensin 119	BNBD119	13	Q32P86	1–20	21–83	27–54, 34–48, 38–55
Bovine β-defensin 127	BNBD127	13	A0A3Q1N9G9	1–22	23–54	23–55, 33–49, 37–56

All defensins undergo a multi-step synthesis process, beginning with a pre-defensin that contains a signal segment, pro-segment and a mature peptide. Their processing varies depending on expression site and typically involves a fast cleavage of the signal peptide of 20 or so amino acids, generating a pro-defensin (Fig. 2). The pro-fragment is thought to promote pro-defensin charge balance, helping to reduce the toxicity of defensin toward eukaryotic cells.^19^ β-defensin has a shorter pro-segment than α-defensin. It may be due to differences in the transcription patterns (α-defensins are usually constitutively produced, while most β-defensin expression occurs in response to stimuli^15^), leading to different processing and intracellular transport requirements for the mature peptides to rapidly react to immune responses. It is worth noting that crystal structure analyses of defensins show that defensins exist as dimers or multimers.^90–92^ Lu et al. have preliminarily studied the importance of dimerization for the biological roles of defensins. They found that these polymers have stronger antibacterial and membrane destruction activity and can enhance binding to multiple molecular targets compared to monomers.^91,93–95^

Based on the difference in the coding exons, α-defensins are classified into myeloid and enteric α-defensins (Fig. 2a). HNP1-4 are four of the six known myeloid α-defensins and are expressed primarily in the granules of neutrophils^96^ and certain lymphocytes,^97^ as well as natural killer (NK) cells.^98^ Notably, mouse neutrophils lack defensins.^99^ HNP1-4 are stored in the azurophilic granules as fully processed mature peptides.^34,100^ Upon fusing with phagasomes, α-defensins-laden azurophil granules then release large amounts of HDPs in the proximity of the pathogen surface, where they quickly penetrate the cell membrane due to their amphipathic nature.^64,101^ The other two α-defensins, HD5 and HD6, are enteric α-defensins that are mainly expressed by Paneth cells (PCs).^102–105^ Unlike pro-HNP1-4 processing and vesicle storage, HD5 and HD6 are stored in secretory vesicles as a pro-peptide and are processed by one or more isoforms of Paneth cell trypsin.^32^ However, whether the pro-HD5 peptide is converted into its mature form during secretion or within the lumen is unclear. In addition to PCs, the reproductive tract and oral cavity also express HD5 and HD6. Interestingly, these two peptides are functionally different. The antibacterial activity of HD5 is to kill bacteria directly,^106^ while HD6 does so indirectly by forming self-assembled nanonets in order to trap bacteria and prevent infection.^7,107–109^ Although mouse neutrophils lack defensins, mouse PCs express more than 20 α-defensins throughout the mouse small intestine,^110–112^ which are also called cryptdins. Seventeen cryptdins (Cryptdin1-17) have been identified at the protein level.^113,114^ All the peptides have potent in vitro bactericidal activity,^41^ with *S. aureus* appearing to be more susceptible to cryptdin-mediated killing than *E. coli*.^41^ Mouse cryptdins are processed into their active form by matrix metalloproteinase 7 (MMP7) during granulogenesis.^6,33^ Indeed, mice lacking MMP7 cannot process the precursors of pro-cryptdin, leading to a deficiency of mature cryptdins, thus impairing their ability to scavenge infections and regulate immune homeostasis.^6^ In mouse and human PCs, mature α-defensin is oxidized to prevent internal digestion.^115^

Compared with α-defensins, the localization of the cysteine residues along the amino acid sequence of β-defensins (BDs), the folding pattern of the peptide chain and the disulfide bond pattern are entirely different (Fig. 2b). The peptide chains of BDs fold to form three β-lamellae with four conserved glycine, proline, threonine and lysine residues. The synthesis and secretion of BDs are also different from α-defensins. BDs are directly secreted into the extracellular space in their mature form to exert immunomodulatory and antibacterial activities.^116^ BDs mainly display stimulated expression, but constitutive expression patterns also exist. For example, the promoter of DEFB1 does not contain response elements for NF-κB and AP-1, so DEFB1 gene expression is not upregulated in response to inflammatory factors but is physiologically expressed in epithelial cells.^117^ However, the expression of most BDs is limited to specific tissues or epithelial cells where they perform a particular function. For example, the production of macaque BD126 is confined to the epididymal epithelium, where it is attached to membranes of sperm cells as they traverse through the epididymis. This exclusive function safeguards macaque sperm from being attacked by the immune system within the female reproductive tract.^118^

Presently, the progress in studying θ-defensins is relatively slow compared with α-defensins and β-defensins. However, from an evolutionary perspective, it is clear that θ-defensin genes arose from mutated α-defensin genes.^30,64^ θ-defensins are the only cyclic peptides in animals (Fig. 2c) and have been isolated from rhesus macaques and baboons. Rhesus θ-defensins (RTDs) are primarily synthesized in the bone marrow and secreted by neutrophils, PCs and monocytes.^119^ Intriguingly, θ-defensins are chimeras of 18 residues formed by spliced heads and tails from two separate precursors, each of which contains nine amino acids.^30,120^ In humans, the θ-defensin gene has an early termination codon that hinders efficient translation of the desired precursor,^121,122^ indicating that θ-defensins are not exist in the human body and were most likely phased out by natural selection.

## Antimicrobial mechanisms of defensins

**Fig. 3:**
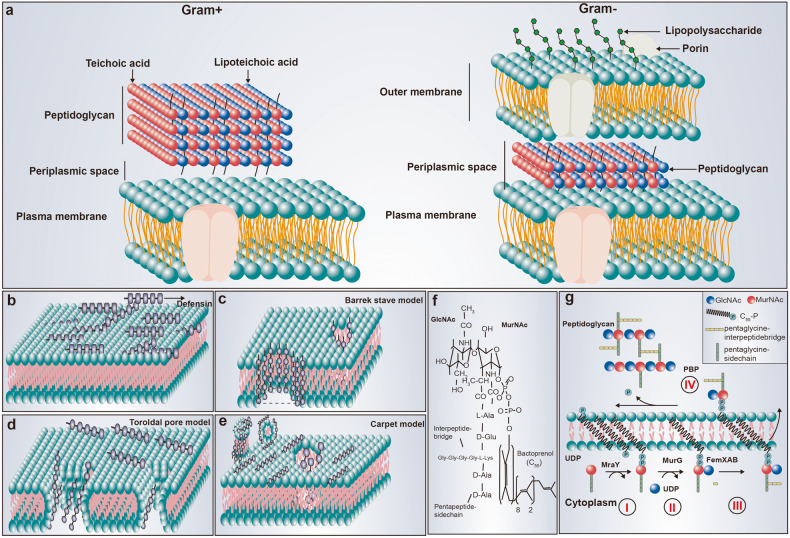
Antimicrobial mechanisms of defensin. **a** The cell membrane structure of G^−^ and G^+^ bacteria. **b** Defensins accumulate on the cell membrane before destroying it. **c**–**e** Illustrations of the various modes of defensins-mediated cell killing, including the barrel-stave model, the toroidal pore model and the carpet model. **f** The structure Lipid II; **g** Cell wall biosynthesis begins in the cytoplasm where UDP-MurNAc-pentapeptide is formed. This soluble precursor is then linked to the membrane carrier bactoprenolphosphate (C_55_P) by MraY, yielding Lipid I (reaction I). MurG subsequently adds GlcNAc to form Lipid II (reaction II). After the formation of the interpeptide bridge (as seen in reaction III), the monomeric peptidoglycan unit undergoes translocation across the cytoplasmic membrane for incorporation into the cell wall (reaction III). It is noteworthy that this interpeptide bridge formation is limited to some Gram-positive bacteria, as highlighted by research.^38^ Note: To better demonstrate the crosstalk mechanism of defensins in regulating immune homeostasis, the intestine containing PCs and mucosal structures was used as the background of the regulatory network

Defensins possess wide-ranging antibacterial activity against both Gram-negative (G^−^) and Gram-positive (G^+^) bacteria in vivo and in vitro.^123–130^ For example, the anti-*Staphylococcal* and anti-*E. coli* activity of hBD3 is 1 mg/L and 4 mg/L, respectively.^131^ However, the cell membrane structure of G^−^ and G^+^ bacteria differ (Fig. 3a) as the cell membrane of G^−^ bacteria has three layers that include an outer membrane, a peptidoglycan layer and a plasma membrane, whereas G^+^ bacteria have only a peptidoglycan layer and a plasma membrane. G^−^ surfaces contain many lipopolysaccharides (LPS) with a negative charge.^132^ By interacting with negatively charged components on the surface of G^−^ bacteria, defensins destroy membrane barrier function. With the accumulation of defensins on the membrane (Fig. 3b), the electrostatic attraction and penetration of defensins bound to the membrane are enhanced, and the defensins freely diffuse and preassemble on the membrane surface,^133–135^ followed by hydrophobic interactions between the amphipathic peptide domain and the membrane phospholipids.^136,137^ There are three primary models for defensin-mediated transmembrane pore formation, which are barrel-stave, toroidal pore, and carpet models.^135,138–141^ The first proposed mechanism for permeabilization was the barrel-stave model, which serves as a prototype for defensin-mediated transmembrane pore formation. Defensins serve as staves that insert themselves vertically into the phospholipid bilayer, yielding barrel-like structures (Fig. 3c), such as HD5 for G^−^ bacteria.^128,142–144^ The toroidal pore model depicts the insertion of defensins into the membrane, causing a consistent curvature of the phospholipid monolayer from the upper portion to the lower portion (Fig. 3d). In the carpet model, peptide-induced membrane disruption is similar to that of a detergent-like action (Fig. 3e). For example, the cell membrane adsorbs hBD3 through strong electrostatic interaction of Arg12 with POPG lipids in G^+^ bacteria.^145^

However, the membrane destruction model cannot fully explain the complete mechanism behind the defensin-mediated bacterial killing. Specifically, it is difficult for this model to account for how defensins can swiftly eradicate bacteria in the LPS-deficient outer membrane of G^+^ bacteria. Thus, it is likely that another mechanism exists for defensin-mediated bacterial killing. One possible alternative mechanism is that defensins disrupt cell wall synthesis (Fig. 3f, g) by targeting the membrane-anchored cell wall precursor, lipid II, which is crucial for the process.^146–149^ Plectasin, a fungal defensin secreted by *Pseudoplectania nigrella*, displays strong antibacterial activity against G^+^ bacteria, even against otherwise resistant clinical isolates.^38^ Tanja and colleagues found that plectasin does not cause any disruptions to membrane integrity as it had no influence on the typical features of the membrane penetration mechanism, such as membrane potential and intracellular K^+^ contents.^38^ Interestingly, plectasin treatment led to an accumulation of the cell wall precursor, UDP-MurNAc-pentapeptide.^38^ Plectasin effectively prevents the interaction between the lipid I and lipid II carriers and cell wall biosynthetic enzymes by bonding with them in a 1:1 molar ratio. The equilibrium-binding constants for lipid II and lipid I are 1.8 × 10^−7^ mol and 1.1 × 10^−6^ mol, respectively, indicating that the second sugar in lipid II, N-acetyl glucosamine (GlcNAc), plays a role in stabilizing this complex.^38^ In addition to plectasin, researchers have identified other defensins targeting lipid II, such as hBD3 and HNP1, Cg-Defh1-2 from crassostrea gigas, oryzeasin and eurocin from fungi, and lucifensin from maggots.^146,147,150–153^ For example, in *S. aureus* treated with hBD3, the UDP-MurNAc-pentapeptide, a cell wall precursor, was also found to accumulate,^147^ like in the case for plectasin. Further, hBD3 was shown to inhibit the activity of staphylococcal penicillin-binding protein 2 (PBD2) when the molar ratio of hBD3 to lipid II is 2:1. However, hBD3 treatment also resulted in a decreased membrane potential, and transcriptome data indicated that hBD3 treatment was partially like HDPs treatment exposed to membrane-active α-helices.^147,150^ Thus, hBD3 exhibits a pleiotropic antibacterial mechanism against *S. aureus* involving cell wall synthesis inhibition via targeting lipid II and effects on membrane permeabilization.

Recently, the Wehkamp lab found that in a reduced physiological environment, the disulfide bridges characteristic of defensins become disrupted, rendering them susceptible to protease degradation. This process liberates novel antimicrobial peptide fragments that enhance the antimicrobial repertoire and may thus be an evolutionary trait enabling the host to mount an effective broad-spectrum response towards invading pathogens with minimal resources. For example, duodenal fluid- and gastrointestinal-derived trypsin degrade full-length HD5, hBD1, and HNP4 into various bioactive fragments with different antibacterial properties. Other fragments showed different antibacterial activity. As an example, HNP4_1-11_ exhibits superior antimicrobial potential in comparison to the intact peptide on mass and molar levels.^154^ Other fragments, including HD5_1-9_, HD5_1-13_, HD5_7-32_, and HD5_fl_, substantially affected the growth of all tested bacterium, while others, like HD5_14-32_ and HD5_10-27_, were ineffective against the tested bacteria under the same experimental conditions.^107^ The minor differences in fragment sequences of HD5_1-9_ and HD5_1-13_ resulted in different antimicrobial activity.^107^ These results suggest that defensin fragmentation is a fine-tuning mechanism for host-microbe interactions.

## The biological functions of defensins

As the number of studies of defensins increases, it has been found that these molecules act in numerous biological processes, including showing immunomodulatory and chemotactic activities, maintaining mucosal barrier function, balancing the gut microbiota and regulating organ development and cell death. Therefore, gradually defensins have been perceived to be innate immune factors. Here, we review the biological functions of defensins that have been discovered to date.

### Immunomodulatory activity

**Fig. 4:**
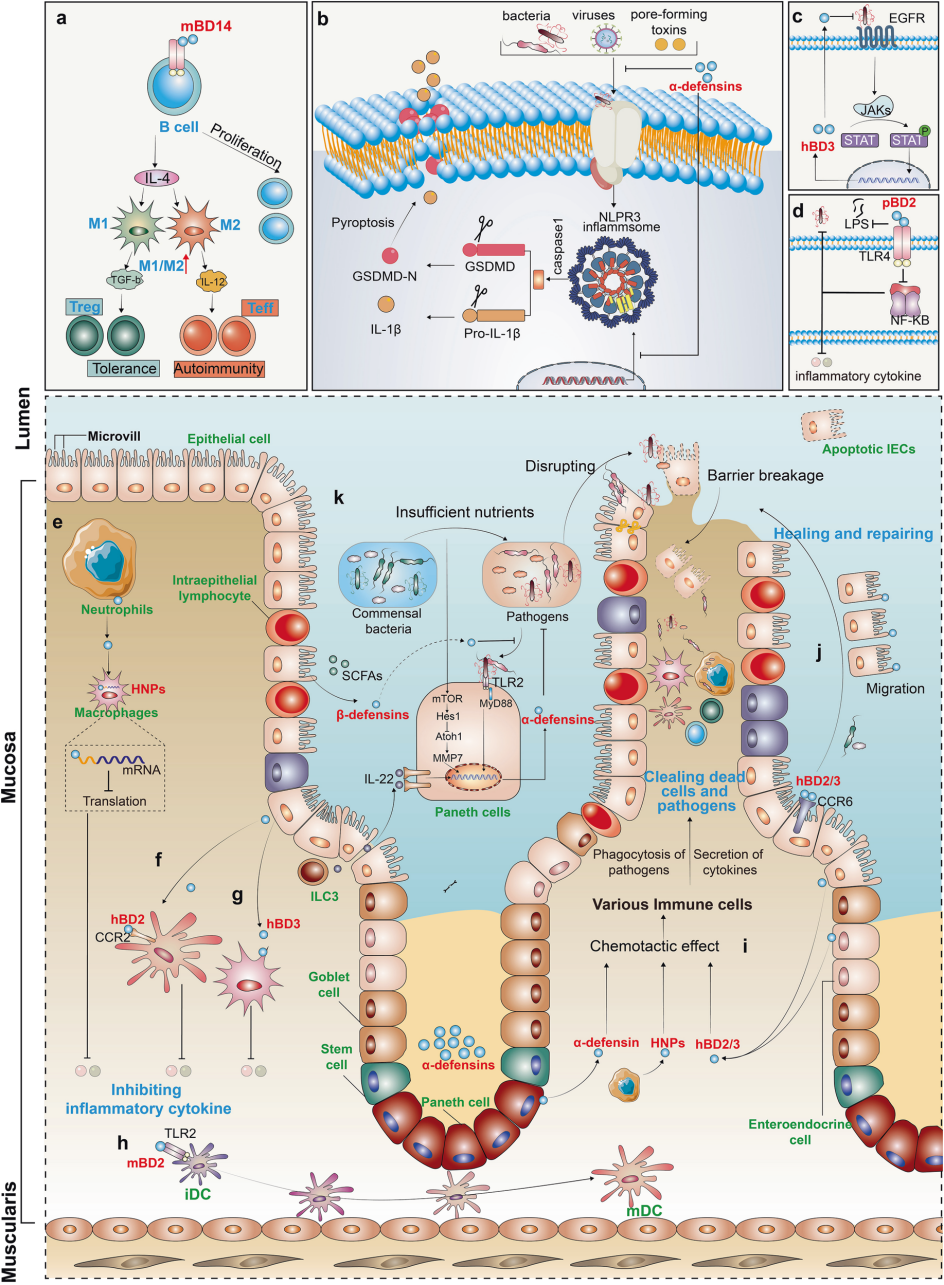
Regulation role of defensins in immune homeostasis. **a** mBD14 promotes B cell proliferation via TLR2 and improves the M1/M2 macrophage balance and induces regulatory T cells. **b** Mature α-defensins prevent NLRP3 inflammasome activation and the release of IL-1β. **c** hBD3 is activated by EGFR-mediated MAP kinase and JAK/STAT signaling pathways after *H. pylori* infection. **d** By competitively inhibiting the LPS-induced activation of the NF-κB via TLR4, pBD2 can effectively restrict downstream inflammatory cytokine secretion. **e** HNP1 released by neutrophils enters macrophages to bind to mRNA, and then inhibits mRNA translation of various inflammatory factors. **f**, **g** hBD2, hBD3, and HNPs inhibit the secretion of inflammatory cytokine; **h** mBD2 promotes the maturation of DCs via TLR4 signal. **i** Defensins recruit various immune cell to clear out dead cells and pathogens. **j** hBD2 and hBD3 regulates the repair of barrier function via the CCR6-*Rho*-ROCK signaling pathway. **k** In a nutrition-deficient state, the continuously activated α-defensins promote the resistance to invasion by enteric pathogens through an mTOR-*Hes1-Atoh1*-MMP7-α-defensins axis

Increasing evidence indicates that the direct bactericidal activity of defensins in regulating the antibacterial immune response is not the only essential role of defensins in regulating host immune homeostasis. Specifically, they also modulate both innate and adaptive immune responses as immune regulatory factors.^1,14,101,155^ Not surprisingly, dysregulation of defensins expression is associated with autoinflammatory and autoimmune diseases, including sepsis, irritable bowel syndrome (IBS), atherosclerosis, thrombosis, rheumatoid arthritis and type 1 diabetes.^74,76,156–162^ However, the involvement of defensins in immune regulation is very complicated, and their role goes far beyond simply acting as immunomodulators via a singular receptor or linear signaling within the immune system (Fig. 4). A case in point of the complex roles of defensins in the immune response is the protein–protein interaction network of hBD3. Notably, hBD3 interacts with no less than 46 proteins or receptors and 1779 genes show differential expression upon hBD3 stimulation of TLR4 agonist KDO2-lipid A-primed mouse macrophage cells.^163^ These varied responses suggest that defensins exert their effects mainly by interacting or trans-activating various extracellular and intracellular receptors.

Regulation of autoimmunity is one of the main functions of defensins. Miani et al. found that endocrine cell-expressed mBD14 promotes B cell proliferation and increases their secretion of IL-4 by acting on TLR2 (ref. ^159^). Subsequently, IL-4 further improves the M1/M2 macrophage balance and induces regulatory T-cell responses to prevent autoimmune diabetes^159^ (Fig. 4a). In addition, mBD2 functions as an endogenous TLR4 ligand that acts upon immature dendritic cells (iDCs), resulting in the enhanced expression of costimulatory molecules and the maturation of DCs^50^ (Fig. 4h). These findings indicate that defensins can regulate acquired immune responses. Further, in the nutritionally-deficient state, the continuously activated α-defensins promote resistance to enteric pathogen invasion via an mTOR-*Hes1-Atoh1*-MMP7-α-defensins axis^164^ (Fig. 4k). In addition, defensins also regulate the expression of inflammatory factors. Koeninger et al.^70^ found that hBD2 improves disease activity indices and prevents colitis-associated weight loss in three mouse models (dextran sodium sulfate (DSS), 2,4,6-Trinitrobenzenesulfonic acid (TNBS) and T-cell transfer into immunodeficient recipient mice). Furthermore, they found that hBD2 engages with CCR2 on DCs to inhibit NF-κB activity and to promote CREB phosphorylation, thus reducing the expression of inflammatory factors (Fig. 4f). Our previous studies showed that pBD2, a porcine β-defensin, competitively inhibits LPS- and DSS-induced activation of NF-κB signaling via TLR4, thus dampening the secretion of inflammatory cytokines^69,165^ (Fig. 4d). Similarly, Zhang et al. and Lian et al. observed that pBD2 decreases the adherence of *E. coli* to cells and alleviates inflammation via the TAK1-NF-κB pathway.^166–168^ Semple and colleagues found that hBD3 is a strong inhibitor of TNF-α and IL-6 accumulation, two potent pro-inflammatory cytokines (Fig. 4g).^169^ Like β-defensins, HNPs are also released from dying neutrophils during apoptosis or necrosis and effectively suppress pro-inflammatory responses by interfering with the production of nitric oxide and inflammatory cytokines from macrophages.^170^ Neutrophils are the initial and most abundant cells to reach the area of inflammation-induced injury, where they release a large amount of the defensin HNP1 (ref. ^101^). In this study, it was shown that HNP1 acts as a "molecular brake" to limit macrophage-driven inflammation.^101^ Notably, neutrophil-derived HNP1 enters macrophages, where its positive charge and amphipathic characteristic help it bind to mRNA to inhibit the translation of various inflammatory factors rather than affecting mRNA transcription and stability (Fig. 4e). In addition, Shi et al. found that defensin-deficient (*Mmp7*^*−/−*^) mice produce more IL-1β in the colon and cecum and are more susceptible to DSS-induced colitis.^171^ Exogenous supplementation with mature α-defensins, rather than precursor α-defensins or β-defensins, inhibit IL-1β secretion following activation of inflammatory NLRP3 inflammasomes in human and mouse macrophages^171,172^ (Fig. 4b). The data indicate that α-defensins may have a significant role in maintaining gut homeostasis by modulating the expression of IL-1β. However, defensin-mediated regulation of TLR signaling does not necessarily exhibit an anti-inflammatory effect, as they can also potently amplify the immune cell response to bacterial DNA via a TLR-9-mediated pathway, while hBD2 and hBD3 can induce self-DNA condensation into particles that are endocytosed by plasmacytoid DCs, resulting in the activation of TLR-9-dependent IFN-α production.^173,174^ Neutrophil-secreted HNP1-3 can also boost bacterial phagocytosis by triggering macrophages to accelerate their expression of TNF and IFNγ.^175^

Moreover, epigenetics plays a regulating role in the production of defensins. For instance, after deacetylase inhibition, NF-κB is modified by the acetylase p300, which enhances the transcription of *Defb2* in colonic primary epithelial cells while decreasing the potential of harmful inflammatory responses.^176^ Our previous study also found that after enterotoxigenic *Escherichia coli* infection, METTL3, an N6-adenosine-methyltransferase, interacts with the transcription factor FoxO6 and modulates *Gpr161* transcription and subsequent regulation of β-defensin expression.^177^

The critical role of defensins in host defense via their immunomodulatory activity is also well-studied. Various bacteria, including *Vibrio cholerae*, *Bacteroides fragilis*, *Pseudomonas aeruginosa*, different *Pseudomonas* species and *Salmonella enteritidis*, regulate the production of hBD2 (refs. ^178–183^). Mechanistically, this regulation is related to the interaction between bacterial flagellum and TLR5 (refs. ^184,185^). Moreover, hBD2 is induced via Nod1-dependent activation of NF-κB after infection by *Helicobacter pylori*^186,187^ or *P. aeruginosa*.^180^ Similarly, infection with *H. pylori* upregulates the generation of hBD3 by the EGFR-dependent activation of MAP kinase and JAK/STAT pathway^188^ (Fig. 4c). Further, an exciting study revealed the existence of a signaling pathway essential for skin resistance to pathogen infection occurs through the interaction between the epithelium and neutrophils via defensins.^60^ Upon *Staphylococcus aureus* infection, defensins are released by keratinocytes and activate Mrgpra2 receptors on neutrophils, which results in IL-1β and CXCL2 release to promote infection resistance. Disruption of this signaling cascade can lead to immune deficiency and abscess formation.^60^

In addition to the pool of defensins secreted by neutrophils and epithelial cells as a response to infection, antigen exposure also triggers the release of defensins in NK cells and PCs.^189,190^ For example, HD6, released by PCs, blocks enteric bacterial pathogen invasion by ordered self-assembly of microbe-entangling peptide nanonets.^7^ *Mmp7* knockout mice have considerably diminished clearance of *E. coli*^6^ and *Chlamydia trachomatis*^191^ in the intestine compared to parental wild-type mice. Moreover, the production of cryptdin family types and levels is higher in conventionally-raised mice than in germ-free mice.^192^ Mechanistically, the NOD2 signaling pathway is essential for PCs to secrete defensins. After bacterial infection, NOD2 recognizes muramyl dipeptide (MDP) and then activates NF-κB, thus upregulating the transcription of defensins.^193–195^ NOD2-mediated defensin regulation is beneficial in protecting against *Haemophilus influenzae*-induced otitis media.^196^ In addition, *Nod2*-deficient mice show different intestinal microbiota compositions from wild-type mice and increased susceptibility to infection upon challenge with *Listeria monocytogenes*.^197^ These studies demonstrate that NOD2-induced secretion of α-defensin plays a vital role in regulating the composition of intestinal microbiota and defending against pathogen invasion.

The COVID-19 pandemic caused by the severe acute respiratory syndrome coronavirus type 2 (SARS-CoV-2) has drastically affected both public health and the economy. As of November 2022, over 700 million cases and 6.6 million deaths have been reported globally.^198–200^ With respect to anti-SARS-CoV-2 infection, defensins have also been considered as potential therapeutic molecules.^201–204^ For example, HD5 inhibits SARS-CoV-2 S1 binding and thereby prevents pseudovirions entry into enterocytes by competitively binding with angiotensin-converting enzyme 2 (ACE2).^205^ Similar effects were also found for HNP1 and hBD2, but not for hBD5 and hBD6 (refs. ^206–208^). For example, by molecular dynamics simulations and by functional studies, it was found that hBD2 interacts with the CoV-2-receptor binding domain (RBD) and obstructs viral entrance of ACE2-expressing cells.^207^ In contrast, HNP1 inhibits viral fusion but does not affect the binding of the spike RBD to ACE2 (refs. ^206,209^). In silico approaches also suggested that defensins can physically bind spike surface viral protein (Sgp), thus preventing its interaction with ACE2 (refs. ^203,210^). Therefore, this evidence suggests that defensins could target ACE2, Sgp or disrupt the viral membrane. Meanwhile, maternal transmission of defensins can protect fetuses from SARS-CoV-2 infection.^211^ Together, these findings provide substantial evidence that defensins are crucial in safeguarding individuals against various bacterial and viral infections.

In summary, defensins are essential for immune regulation, but many questions remain, including how do defensins, usually considered innate immune factors, activate so many immune pathways? Is the pathway of defensin activation due to an inherent characteristic of the peptides or in response to different immune activation modes? Are they an effector, sensor or activator in immune regulation? Moreover, what are their means of action? Elucidation of these questions is essential if defensins are to become actionable clinical therapeutic targets.

### Chemotactic activity

Chemotactic activity is a vital factor in driving the coordinated migration of immune cells in and out of tissues, as well as dictating their spatial organization and interaction in tissues^212^ (Fig. 4i). Several studies have reported that α-defensins, as well as β-defensins, like chemokines, play essential roles in immune cell activation and recruitment.^54,213^ Moreover, the concentration of chemotactic defensins is lower than that of bactericidal defensins.^214^ The earliest clues to the chemotaxis of defensins were the findings that HNP1 and HNP2 induce migration of human monocytes^215^ and T cells.^216^ Subsequent studies revealed that HNP1 selectively chemo-attract naive T cells and iDCs.^55^ The pre-treatment of pertussis toxin could depress the chemotactic activity stimulated by most defensins, suggesting that this activity depends on Gi-protein-coupled receptors (GPCRs)^20^ (Table 2). It has been reported that hBD2, hBD3, mBD2, mBD3, and mBD29 have chemotactic activity on T cells and iDCs via interacting with the chemokine receptor CCR6 (refs. ^54,217–219^). Interestingly, hBD2 and hBD3 also utilize CCR2 to regulate monocyte and macrophage trafficking.^220,221^ This suggests that some defensins use more than one GPCR to induce cell migration. Moreover, Rohrl et al. also demonstrated that mBD4 and mBD14 interact with CCR2 in monocytes, macrophages, and neutrophils.^221^ A similar phenomenon in β-defensin-1 has also been found in fish.^222^

**Table 2:** Target cells and receptors of defensins-induced cell migration

Family	Defensins	Target cell	Receptor		Reference
			GPCR	Non-GPCR	
α	HNP1	Mo/Mφ, naive T, memory T, iDC, MC	n.d.	n.d.	^55,215,216,482^
α	HNP2	Mo/Mφ, T cell	n.d.	n.d.	^215,216^
α	HNP3	Mo/Mφ, memory T, MC	n.d.	n.d.	^482^
α	HD5	Mo/Mφ, naive T, memory T, MC	n.d.	n.d.	^482^
β	hBD2	Ep, memory T, iDC, MC, Mo/Mφ	CCR6, CCR2	EGFR	^54,221,483–485^
β	hBD3	Ep, Mo/Mφ	n.d.	EGFR	^220,221,484^
β	hBD4	Ep	n.d.	EGFR	^483^
β	BEBD, BNBD3/9	iDC	n.d.	n.d.	^486^
β	mBD2, mBD3, mBD29	iDC	CCR6	n.d.	^217,218^
β	mBD4	Mo/Mφ, DC	CCR6, CCR2	n.d.	^221,487^
β	mBD14	Mo/Mφ, HEK293	CCR6, CCR2	n.d.	^221,488^
β	maBD1	Mo/Mφ	n.d.	n.d.	^222^

*CCR* CC chemokine receptor, *EC* endothelial cell, *Ep* epithelial cell, *Mφ* macrophage, *Mo* monocyte, *n.d.* not determined, *BEBD* bovine enteric β-defensin, *BNDB* bovine neutrophil β-defensin

Chemotaxis of defensins facilitates the flow of inflammatory effector cells and effector molecules to the site of infection, enabling the body to kill pathogenic microorganisms more effectively while providing a bridge between natural and acquired immune responses.^223^ However, the mechanism of β-defensin’s chemotactic action is better understood than the chemotactic properties of α-defensins, as currently, the receptors responsible for mediating the chemotactic effects of human α-defensins have not been characterized.

### Maintaining the mucosal barrier

The mucosal barrier is the initial line of defense. Thus, rapidly promoting the repair and reconstruction of mucosal damage is especially important for organisms to maintain homeostasis. The breakdown of barrier function leads to Crohn’s disease (CD) and atopic dermatitis (AD).^224–228^ In the past, for both ileal and colonic CD, the absence of defensins was thought to be only associated with a general reduction in mucosal antibacterial activity.^224,229^ However, presently, studies have found that defensins can repair barrier damage by promoting epithelial cell proliferation. Moreover, they also actively participate in controlling the expression of barrier-specific proteins to maintain barrier function.^224,226,229,230^ For instance, in the cuticle barrier of the skin, hBD1 and hBD3 through CCR6-aPKC-Rac1 and CCR6-GSK3-PI3K signaling increased the expression and cell membrane positioning of barrier proteins. This leads to elevated trans-epithelial electrical resistance and reduced permeability in keratinocyte layers.^226,231^ In the intestine, hBD3-induction not only promotes intestinal epithelial cells (IECs) migration and preserves the intestinal barrier through CCR6-Rho-ROCK (Fig. 4j) but also inhibits autophagy through the CXCR4 signaling pathway, which significantly promotes IECs migration and maintains mucosal integrity.^232–234^ In addition, hBD2 can stimulate migration, proliferation, and tube formation in colonic epithelial and endothelial cells, thereby accelerating the closure of wounds.^235–238^ Mechanistically, Koeninger et al.^70^ found that hBD2 engages with CCR2 on DCs, which leads to a reduction in NF-κB and an increase in CREB phosphorylation, ultimately reducing inflammation. Of note, hBD2 has been employed as an indicator of disease severity and skin barrier characteristics in human allergic dermatitis and tinea corporis diseases.^239,240^ These findings indicate that the function of β-defensins in promoting the mucosal barrier primarily depends on activating the chemokine receptor family.

Like β-defensins, α-defensins also play an essential function in maintaining the mucosal barrier. In a mouse model, an increase in heat stress results in the upregulation of cryptdin2 expression. In addition, the severity of the heat stress-induced injury to intestinal barrier function positively correlates with the levels of cryptdin2 in both serum and the intestine.^241^ In humans with liver cirrhosis, compromised HD5 and HD6 function inhibits the function of T cells. Subsequently, immune cell deficiency perpetuates the vicious cycle of inflammation, causing elevated intestinal permeability as well as bacterial translocation.^242^ In patients with CD, TCF1-, and TCF4-mediated regulation of Wnt signaling-driven HD6 secretion by PCs is disrupted, which damages the repair of the mucosal barrier.^104,243,244^

Surprisingly, defensins can also be negatively regulated by the mucosal barrier. The epidermal growth factors (EGFs), essential for wound repair, can induce the expression of hBD3 after epidermal cell wounding.^245^ TGF-α, a member of the EGFs, participates in the repair process after mucosal damage.^246^ When the mucosa is injured, the expression of TGF-α increases rapidly. TGF-α can promote the proliferation of PCs and crypt cells in vivo, which secrete many defensins that maintain immune homeostasis as indicated by the repair of intestinal mucosa and wound healing.^246,247^ A defective MUC2 mucin barrier, typical in IBD, leads to deficient stimulation of hBD2 and barrier repair.^248^

Although recent studies and their conclusions, without exception, describe HD5 as a critical molecule in the human gut that fights off microbes and inhibits damage, a recent study provided the opposite conclusion. It showed that HD5 promotes the adhesion of *Shigella* to destroy the epithelial barrier function by targeting bacterial membrane proteins and that this process depends on the native tertiary structure and the critical residue of Arg28 of HD5 (ref. ^249^). This finding fundamentally challenges the understanding of the role of defensins as “protectors”, which may be due to the unique properties of HD5 and *Shigella*, or that *Shigella* has possibly evolved to highjack this function of HD5.

### Balancing the gut microbiota

It is known that the gut microbiota is a highly complex ecosystem that performs crucial physiological functions, including maintaining intestinal barrier integrity, promoting immunological fitness, and maintaining metabolic homeostasis, and that it dynamically responds to intrinsic and extrinsic stimuli. The microbiota community in humans comprises ~1000 species, involving up to 10^15^ procaryotic cells, with a weight of 1 kg and a ratio to eukaryotic cells that is approximately 1:1 (refs. ^250,251^). In recent years, it has been found that HDPs, especially defensins, are crucial for intestinal homeostasis and recovery of intestinal microbiota.^252–254^ For example, PCs directly sense the presence of gut commensals, and they preserve homeostasis of the intestinal-microbial interface by secreting several members of the α-defensin family.^255^ A new study has provided novel insight into how gut bacteria interact with defensins to prevent non-obesity diabetes (NOD).^159^ The pancreatic endocrine cells of NOD mice showed almost no expression of mBD14, and treatment with mBD14 significantly reduced autoimmune responses and the incidence of diabetes from 85% to 35% in NOD mice.^159^ Compared with naive NOD mice, the production of mBD14 was significantly upregulated in NOD mice receiving gut microbiota from normal mice.^159^ Further studies showed that the aromatic hydrocarbon receptor ligand and butyric acid, products of the gut microbiota, can facilitate the secretion of IL-23 and IL-22 through innate lymphoid cells (ILCs) of the pancreas, and the latter triggers the transcription and secretion of mBD14 in pancreatic endocrine cells.^159^

**Fig. 5:**
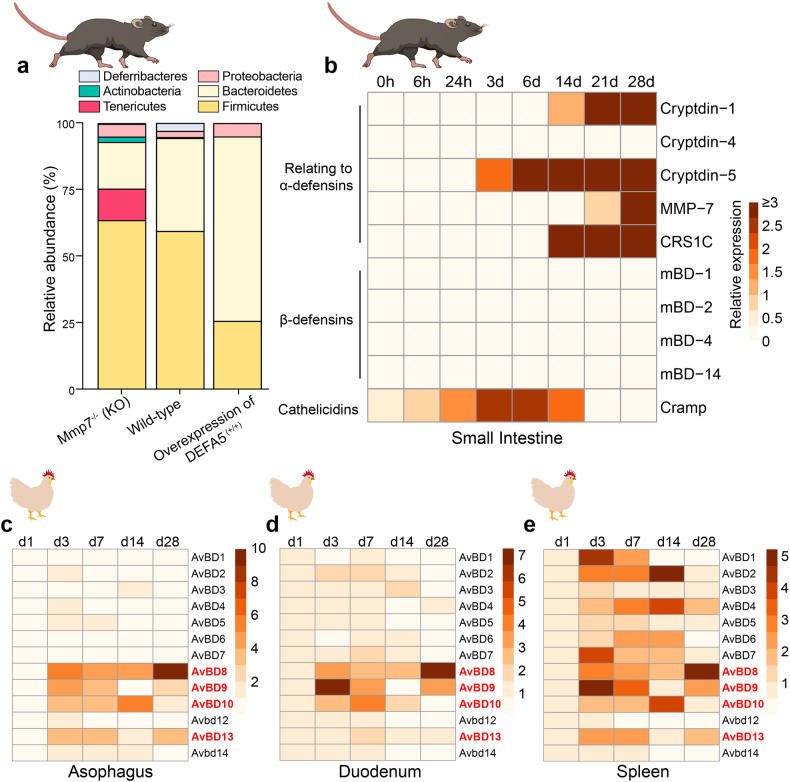
Regulation role of defensins in gut microbiota and intestinal development. **a** Intestinal microbiota composition in HD5 and Mmp7 transgenic mice. **b**–**e** Defensins gene expression maps, including for the small intestine of mice during 0–28d after birth, the esophagus of chicken during 1–28d, the duodenum of chicken during 1–28d and the spleen of chicken from 1 to 28d

Germ-free and gene-deficient animals are essential tools for studying the function of gene coding and the interaction between organisms and microorganisms.^256^ For example, by gene editing, Salzman et al. constructed Mmp7 and HD5 transgenic mice. Their research revealed that Mmp7 and HD5 does not affect the total bacterial numbers; however, there is a reduction in the population of *Firmicutes* and a corresponding enhancement in *Bacteroidetes* in HD5^+/+^ mice compared with wild-type mice. In the Mmp7^−/−^ mice, they found an opposite change. We re-analyzed their 16 S ribosomal RNA sequencing data and found that the abundance of *Firmicutes* increases with the loss of active defensins, whereas the number of *Bacteroidetes* decreases proportionally, and other mechanisms are responsible for maintaining bacterial numbers^257^ (Fig. 5a). Further, they found that defensin deficiency significantly increased segmented filamentou*s* bacteria (SFB) colonization in *Mmp7* knockout mice. The mice overexpressing HD5 exhibited opposite results, which are associated with the level of lamina propria Th17 cells.^257^ This provides evidence that defensins can activate acquired immune responses via controlling the intestinal microbiota.

Not only has the ability of defensins to regulate the composition and metabolic function of intestinal microbiota been directly demonstrated through gene deletion in mice, it has also been found to be true in a human clinical correlation study and a mouse defensin feeding experiment. For example, lower HD5 secretion in older adults compared with middle-aged people is linked to age-related differences in gut microbiota composition.^258^ Specifically, the study identified a negative correlation between the fecal concentration of HD5 and *Alistipes* and *Christensenellaceae R-7*. Previous studies have shown that *Alistipes* has pathogenic effects on colorectal cancer and is associated with symptoms of depression.^259^ Furthermore, *Christensenellaceae R-7* has a negative correlation with body mass index in various populations and its presence increases with age.^260^ These findings suggest that low HD5 may contribute to age-related differences in gut microbiota and increase the risk of disease in older adults. In addition, rebamipide, a drug used to protect the gastrointestinal mucosa, has been found to have the ability to regulate the small intestinal microbiota. Specifically, it can upregulate α-defensin-5 in the small intestine while simultaneously downregulating the presence of *Bacteroides* and *Clostridium*, while upregulating *Lactobacillus*, thereby inhibiting indomethacin-induced small intestinal injury.^261^

Dysbiosis, which refers to the imbalance of the intestinal microbiota composition, is associated with psychological stress and has been known to trigger or worsen symptoms of depression. Psychological stress-induced reductions in α-defensin-5 levels result in microbiota dysbiosis in mice with depression, and α-defensin-5 supplementation attenuates the unbalanced gut microbiota and metabolites.^262^ Fecal α-defensin-5 concentrations have been significantly correlated with gut microbiota composition, including being positively correlated with the beneficial bacteria *Ruminococcaceae*, *Allobaculum*, *Sutterella*, and *Akkermansia*, but they have been negatively correlated with the harmful bacteria *Erysipelotrichaceae*.^262^ In addition, a recent study reported that hBD2 ameliorates acute graft–versus–host disease (aGvHD) through regulating the gut microbiome to limit ileal neutrophil infiltration and restrain T-cell receptor signaling.^263^ Tamima and colleagues found that induction of hBD2 is impaired in cases of aGVHD in both humans and mice. However, when hBD2 was administered, the severity and mortality of aGVHD were reduced. This can be traced back to hBD2’s effect on the intestinal microbiome, specifically an increase in multiple *Bacteroides* species and a reduction in *Ruminococcaceae*. These changes coincide with a reduction in neutrophil recruitment into the ileum of aGVHD mice. Interestingly, studies have demonstrated that an increase in *Bacteroides* is linked with lower GVHD severity in mice.^264^ It is essential to acknowledge that the decreased neutrophil infiltration in the ileum that results from hBD2 treatment was reversed when antibiotics were given to the mice. Thus, the data suggest that hBD2’s effects on intestinal neutrophil infiltration are dependent on intact microbes. In conclusion, hBD2 not only alters the composition of specific intestinal microbiomes, but it is also a critical factor in treating GVHD.

In summary, these studies indicated that PCs and epithelial cells in the intestinal mucosa establish a host immune barrier by secreting defensins, thereby improving the host’s ability to maintain a commensal relationship with microorganisms while allowing an appropriate response to changes in the gut microbial population throughout the host’s life cycle.

### Regulation of intestinal development

The intestinal tract is a key site for nutrient digestion and absorption and has gradually been regarded as the largest immune organ. Healthy intestinal development is principally related to the normal functions of all organs and tissues of the body. Internal factors that affect intestinal development include hormone homeostasis, nutrient metabolism, growth factors, and immune effectors. In recent years, it has been found that HDPs, especially defensins, are also closely related to intestinal development. Although existing research suggests that fetuses are in a non-sterile environment, all mammals have far fewer gut microbial communities before birth than after, and the gut microbiome immediately changes after birth as breast milk and other nutrients are ingested.^256,265^ Collado et al. found that the microbial content of breast milk remains stable over time.^266^ Likewise, the intestinal microbiome gradually stabilizes,^267^ and by adulthood it is composed of an established climax community that is chiefly marked by obligate anaerobes.^268,269^ The expression of HNP1-3 increases with age in one- to three-year-old children, in parallel with their growing microbiome colonization.^270^ The gut microbiota stimulates ILCs to secrete mBD14 to prevent underdevelopment diseases.^159^ In intestinal development, PCs physically appear 2 weeks after birth, whose formation and maturation depend on Wnt signals.^271^ Van et al. found that TCF4 deficiency inhibits PC maturation and epithelial cell proliferation in mice, thus inhibiting the expression of cryptdin1 and cryptdin6 (ref. ^271^). In addition, an exciting study investigated the dynamic pattern of HDPs during the earliest stages of small intestine development.^272^ Here, we show the dynamic expression map of HDPs by analyzing their data (Fig. 5b). The data shows that the expression of defensins and related genes could not be detected in newborn mice, whereas they continuously express cathelicidins. By 21 days after birth, the IECs no longer express cathelicidins. The reduced expression of cathelicidins first occurs in the crypts and lower villi and reaches the tip of the villi some days later. In contrast, the expression of α-defensin and its related genes begin at 14 days after birth, which is associated with PC maturation.^272^ Menard et al. found that global knockout of *Cramp*, which encodes for cathelicidin, promoted the maturation of PCs, as well as IEC proliferation, in mice.^272^ These results suggest that cathelicidins maintain intestinal health at the earliest stage of development, while the function of defensins in maintaining intestinal health starts from day 14 after birth. Moreover, when cathelicidins expression is inhibited, PCs mature several days ahead of time, which initiates the expression and secretion of defensins, thus maintaining intestinal development. The developmental expression of HD5 and HD6, human defensin family members, has also been confirmed, where their mRNA levels tend to be lower during fetal life as compared to newborns and adults.^273^ Notably, it has been demonstrated that the expression of intestinal defensin mRNA during the second trimester of pregnancy is substantially lower, ranging from a 40 to 250-fold difference compared to the levels detected in the adult gut.^274^ Although BDs (Ct > 30) are less expressed in the intestine than α-defensins, in chicken it has been shown that during the first month of development, the spleen is the predominant site of BDs expression.^275^ By re-analyzing their data, we demonstrated the dynamic expression of β-defensins (avian β-defensin, AvBD) in different chicken organs (Fig. 5c–e). The results showed that most β-defensins were still low in expression in the esophagus and duodenum, while most β-defensins were highly expressed in the spleen.^275^ At the same time, AvBD8, AvBD9 and AvBD10 showed similar high expression in the three tissues, indicating that these three β-defensins may serve as critical regulators of tissues and organ development in chicken.

These data suggest that newborn intestinal epithelium lacks complete enteric defensins, and development regulates their expression. However, current research has underestimated the importance of HDPs, especially defensins, in intestinal development. Unfortunately, the precise mechanism behind this phenomenon is still unclear, though it may be linked to either the maturation of PCs or the intestinal microbiota. Whether the HDPs are directly related to the homeostasis and/or the development of the gastrointestinal tract, and is part of the inherent mechanism of such, remains to be further studied.

### Regulation of cell death

Unlike bacterial cell membranes, eukaryotic cell membranes are rich in amphiphilic molecules. Negatively charged phospholipids are predominantly present on the cytoplasmic side, while amphiphilic phospholipids are predominantly distributed on the extracellular (or organelle) side. This results in a neutral charge of the overall eukaryotic cell membranes.^276–278^ Mostly, defensins are not cytotoxic to most eukaryotic cells. Even so, in some situations, recent evidence has shown that defensins are involved in several cell death pathways, such as apoptosis, pyroptosis and necrosis. For example, high-concentration HNP1 enters human bronchial and alveolar epithelial cells, where they quickly translocate to the endoplasmic reticulum and activate caspase-3, the main executioner of apoptosis.^279^ HNP1 also promotes alcohol-induced hepatic fibrosis and hepatocyte apoptosis.^280^ However, HNP1 can inhibit the apoptosis of neutrophil cells through P2Y6-mediated Bcl-xL and caspase-3 and decrease mitochondrial membrane potential.^281^ In addition, it has been discovered that a high concentration of HD5 induce apoptosis in IECs and primary CD4^+^ T cells,^282^ whereas hBD3 can trigger apoptosis and the production of IL-8 in airway smooth muscle cells.^283^ Antigen-presenting cells (APCs), including DCs, monocytes and macrophages, are critical in initiating, modulating and resolving inflammation due to their ability to sense, process and present antigens.^284,285^ HD5 and mBD2, respectively, interact with tumor necrosis factor receptors (TNFR1 and TNFR2) outside the cell membrane and are subsequently translocated to mitochondria, targeting the mitochondrial membrane to induce apoptosis of macrophages and DCs.^286,287^ In addition, hBD1 inhibits apoptosis in DCs through CCR6 and promotes the monocyte differentiation to iDCs and the final maturation of DCs stimulated by LPS.^288^ These findings indicate that defensins have an important immunoregulatory function in controlling the natural process of elimination and maturation of APCs. Defensins have been shown to induce the death of tumor cells.^289–296^ Ninety percent of renal clear cell carcinomas and eighty-two percent of prostate cancers, specifically, lose expression of hBD1 (ref. ^297^). However, the synthesized hBD1 inhibits the proliferation of the bladder cancer cell. In addition, the activation of caspase-3 and consequent cell apoptosis is observed in SW156 kidney cancer cell line when DEFB1 gene is overexpressed.^298^ Jurkat T cells and A549 cells undergo cell death when exposed to HNP1-3, which triggers caspase-3 and caspase-7 activation and ADP-ribose polymerase cleavage in Jurkat cells.^295^ These studies suggest that defensin-induced or -regulated apoptosis may vary depending on the cell type, immune status and defensin concentration. However, the effect of defensin-promoted apoptosis on the host’s innate or adaptive immune response remains unclear.

Beyond their implications in apoptosis, defensins are also involved in pyroptosis and necroptosis. Using HNP1 and HNP3 transgenic mice with neutrophil-specific expression of the defensins, Chen et al. observed that increased gene copy number of HNP1/HNP3 promotes pyroptosis in an NLRP3-dependent manner mediated by P2X7 (refs. ^74,299^). Wang et al. used an LPS-primed macrophage model to demonstrate that hBD2 enhanced IL-1β secretion and pyroptosis, and this is mediated by P2X7-dependent expression of NLRP3.^300^ Ethidium bromide uptake test results, on the other hand, indicated that HNP1-induced P2X7-K^+^ efflux-caspase-1 signaling contributes to pyroptotic pore formation. This suggests that in macrophages HNP1 promotes pyroptosis and IL-1β secretion by acting on various functions of the NLRP3 inflammasome downstream of P2X7 (ref. ^299^). Moreover, studies utilizing double-stranded RNA-induced ablation models have suggested that the ADAM10-Notch signaling pathway strengthens skin innate immunity via enhancing mBD6 expression downstream of type I interferon responses, thereby investigating the relationship between the endopeptidase ADAM10 and pyroptosis of hair follicles.^301^

With respect to necroptosis, research has shown that in atrazine-induced programmed necrosis, as well as immune dysfunction of grass carp hepatocytes, there is a downregulation of β-defensin.^302^ Notably, reduced α-defensin expression and necroptosis of PCs are both associated with ileal CD.^303,304^ This indicates that α-defensin, or perhaps other defensins in the ileum, potentially play a crucial role in the disease through a mechanism related to necroptosis.

In addition, increasingly novel types of regulated or programmed cell death, such as ferroptosis,^305,306^ cuproptosis,^307,308^ parthanatos^309–311^ and lysosome-dependent cell death (LCD),^312,313^ have been discovered. Each of these exhibits distinct molecular cascades and regulatory pathways. However, solid evidence for the specific role of defensins mediating these forms of programmed cell death requires further investigation.

## Clinical relevance and therapeutic potential of defensins

**Fig. 6:**
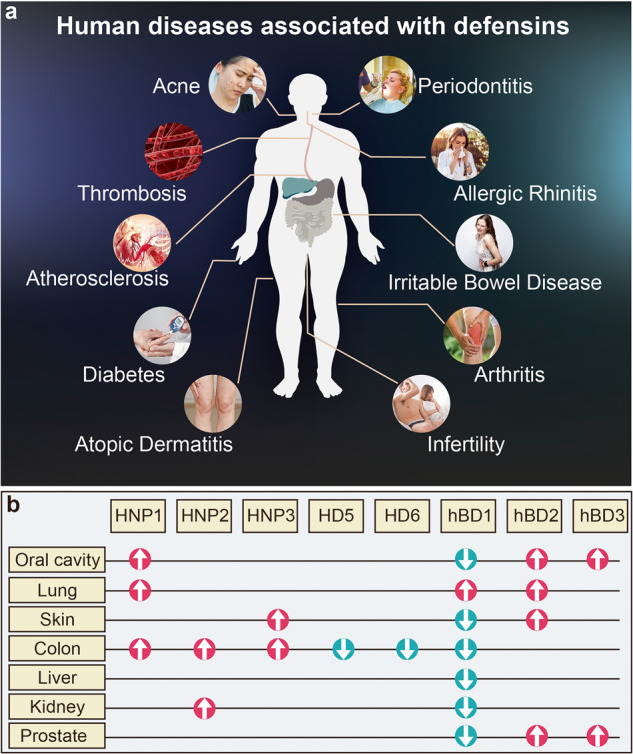
Defensins in disease. **a** Human diseases directly or indirectly associated with defensins. **b** HNP1-3, HD5, HD6, and hBD1-3 are either increased (red arrow) or decreased (aqua arrow) in cancers from different anatomical locations within the human body

The function of defensins in immune regulation has been discussed above. Therefore, it is of great research value for biomedical investigators to use defensins and their derived peptides as a basis to develop and test new therapeutics to treat both infectious and autoimmune diseases. As a starting point for this goal, basic research into defensins over recent decades have resulted in the identification of 348 defensins from animals, plants, and microorganisms, which together provide a sturdy groundwork for further translation of the field. Some of the advances based on the role of defensins in disease pathology (Table 3 and Fig. 6a) and the formulation of therapeutic strategies for defensins or their derived peptides designed based on defensins (DPDs) are summarized below.

**Table 3:** Various human diseases associated with defensins

Disease	Defensins	Level	Defensins mechanism in disease	Reference
Periodontitis	hBD1-2	Low	Low levels of hBD1-2 are associated with periodontitis.	^489–491^
Periodontitis	HNP1-3	High	The pathogenesis of severe periodontitis may be aided by a local deficiency in HNP1-3.	^385,386^
Periodontitis	hBD3	High	By suppressing inflammatory responses in macrophages, hBD3 exhibits the potential to hinder the progress of periodontitis.	^383,387^
IBD	HD5	Low	Low levels of HD5 disrupt the balance of intestinal microbiota, causing the overgrowth of bacteria and the invasion of potentially pathogenic bacteria into the epithelium. This leads to abnormalities in the intestinal tract’s function.	^303,341,492,493^
IBD	hBD3	High	On the one hand, hBD3 may resist microbial attack on the surface of the intestinal cavity. On the other hand, hBD3 enters the lamina propria and performs chemotaxis to recruit immune cells.	^326^
IBD	hBD2	High	hBD2 reduced inflammation and improved disease activity indices, which may have been due to its impact on the activation of the NF-κB pathway.	^237,324–330^
IBD	HNP1-3	High	HNP1-3 may be a risk gene for severe UC, and its high expression in UC patients may induce an over-immune response, but a low dose of HNP1 can relieve colitis.	^78,340,356–358^
T1MD	HNP1-3	High	By activating a c-Src-dependent signaling pathway, HNP1 can reduce hepatic gluconeogenesis and lower blood glucose levels.	^71,160,364^
Obesity	HD5	Low	HD5 enhances glucoregulation and ameliorates the lipid profiles in both the plasma and the liver.	^72,366^
COPD	hBD1-2	High	hBD2 levels are positively correlated with severity of COPD and IL-8 levels.	^372,374,375^
Vitiligo	hBD1	Low	hBD1, along with its gene polymorphisms, could potentially influence an individual’s vulnerability to vitiligo, as well as the level of disease activity.	^494^
Infertility	hBD1	Low	Male infertility caused by leukocytospermia and asthenozoospermia is associated with hBD1 deficiency. hBD1 is capable of elevating sperm motility and egg-penetrating ability via trigging a CCR6-dependent Ca^2+^ mobilization.	^495,496^
Acne	hDB1-2	High	While defensins are effective against Propionibacterium acnes, their accumulation may lead to the formation of lesions in the epithelial tissue.	^497,498^
Psoriasis	hBD2	High	After exposure to hBD2, Th17 cells were recruited to promote the development of skin pathology in psoriasis patients.	^158,499^
Atopic dermatitis	hBD1-3	Low	Th2 cytokine environment can inhibit the expression of hBD1-3 in AD, which may contribute to increased susceptibility to skin infections and exacerbate the symptoms.	^226,500^
Allergic rhinitis	hBD1-3	Low	Th2 cytokine environment can inhibit the expression of hBD1-3 in AR. The decreased levels of hBD1-3 may increase their susceptibility to respiratory tract infections and exacerbations in the tonsils of these patients.	^501–503^

### Clinical relevance and preclinical studies of defensins

#### Infectious disease and defensins

Although significant progress has been made in understanding the disease-causing nature of pathogens and developing treatments to fight infection, infectious diseases remain a leading cause of death around the world.^314^ In fact, in 2019 alone, they were responsible for over 13.7 million fatalities.^315^ Despite advances in medicine, our current antimicrobials have become less effective over the past few decades due to the increasing prevalence of drug resistance, as exemplified by multidrug-resistant tuberculosis.^314,316^ Notably, the immunomodulatory activity of defensins in clearing pathogenic infections is extensive and challenging for microorganisms to develop resistance to.

Numerous studies have highlighted the therapeutic potential of defensins as a form of treatment for various types of infections. One such example is the prevention of mycobacterium tuberculosis in mice through the subcutaneous injection of HNP1. Moreover, in vitro mechanistic experiments further demonstrated beneficial outcomes to verify using HNP1 as an anti-infective agent for tuberculosis.^317^ Exogenous supplementation of recombinant hBD1 or hBD2 effectively controlled *Salmonella* infection. Nearly 50% of infected mice that were inoculated with recombinant hBD1 or hBD2 were still alive 206 h post-inoculation compared to complete lethality within just 24 h for control mice, while in the liver and spleen, the abundance of live *Salmonella* was remarkably reduced in the treated mice.^318,319^ Deficiency of mBD2, an analog of hBD2, in a mouse model of local *P. aeruginosa*-mediated corneal infection showed a worse outcome than control mice, indicating that mBD2 promotes resistance to *P. aeruginosa*-induced keratitis.^79^ Likewise, synthetic nine-mer peptides, specifically ALYLAIRRR and ALYLAIRKR, developed based on the active fragment of insect defensins, have been observed to provide protection in mice infected with lethal Methicillin-resistant *S. aureus* (MRSA).^320^

Similarly, administering exogenous defensins has also achieved beneficial effects against viral pathogens. For example, HNP4 and HD6 can block herpes simplex virus (HSV) infection.^321^ In addition, studies have shown that recombinant mBD2, when given before or after exposure to human influenza A virus (IAV), can protect experimental mice from a lethal virus challenge by 70% and 30%, respectively.^322^ pBD2 inhibits the proliferation of pseudorabies virus in transgenic mice.^75^ It is worth noting that Zhou Rui’s laboratory constructed the first pBD2 transgenic pig and explored the role and mechanism of pBD2 transgenic pig in swine influenza virus (SIV) infection. Studies have shown that pBD2 transgenic pigs can effectively relieve SIV-related clinical symptoms. Mechanistically, pBD2 enters host cells, mediated by energy-dependent endocytosis, to bind SLC25A4, a pro-apoptotic molecule.^80^ This interaction inhibits SIV-induced cell apoptosis.^80^

These experimental data all confirm the excellent therapeutic potential of defensin in anti-infection. Despite these benefits, no clinical trials currently utilize human defensin molecules in infectious disease treatment. Still, several clinical trials have involved the use of two defensin analogs, which will be discussed later (6.2 Clinical Trials of Defensins).

#### Inflammatory bowel disease and defensins

IBD, including ulcerative colitis (UC) and CD, is a complex barrier disease marked by a loss of tolerance towards commensal microbes, altered microbial composition, barrier dysfunction and chronic inflammation of temporal intensity.^323^ In the intestine, defensins help strengthen host immunity and help maintain the correct balance between defending against harmful pathogens and tolerating beneficial microorganisms. However, when the expression of defensins decreases, it disrupts immune homeostasis and exacerbates intestinal inflammatory response. Therefore, the alteration of defensin expression is considered an indispensable factor in the pathogenesis of IBD.

##### β-defensins: focusing on hBD2

The most replicated finding in active IBD is an increase of hBD2. Patients with UC exhibit a ten-fold increase and patients with colonic CD have a 3–4-fold increase compared to controls, and thus both groups express hBD2 at relatively high levels, especially in the inflamed tissue vs the non-inflamed tissue; however, there was no obvious difference in patients with ileal CD.^237,324–330^ Notably, in UC, hBD2 levels increase with the degree of inflammation, whereas this is not observed in CD.^330^ Another study found that patients with colonic CD exhibit reduced functional antimicrobial activity against commensal gut microbiota compared to patients with UC,^229^ but it is unclear if this difference is hBD2-mediated. The differences in hBD2 abundance observed between UC, colonic CD and ileal CD have different mechanisms. The most pronounced genetic risk factor of CD, especially ileal CD, is a frameshift mutation in the *Nod2* gene (around one-third of patients with CD carry this mutation), rendering them incapable of proper hBD2 expression.^331–334^ In contrast, patients with UC exhibit diminished colonic mucin production, which may prevent hBD2 (and other HDPs) from being chemostatically retained in the mucus layer.^324,330,335^ Thus, enhanced hBD2 expression in UC is likely a counter-response to protect against microbial encroachment caused by diminished barrier function, as well as defects in mucus production, whereas reduced or unaltered hBD2 expression in CD may instead relate to different disease pathology and etiology (such as frameshift mutations).

In addition, hBD2 is distributed differently among the colon cell population. Patients with UC exhibit notably higher hBD2 expression in the luminal/villous compartment (I/v-IEC) compared to the crypt compartment (c-IEC), suggesting that mature IECs facing the intestinal lumen are responsible for producing more hBD2 (ref. ^328^). The production of defensin by plasma cells is also thought to be clinically relevant in UC since these cells accumulate in large numbers between the distorted crypts and muscular mucosae.^336^ According to Rahman et al., there is a significant increase in plasma lineage cells observed in colonic samples of patients suffering from UC compared to those with CD and control patients, and hBD2 secreted by plasma cells was upregulated by two- to threefold.^336^ This highlights the potential mechanism by which plasma cells regulate UC through hBD2 at sites of intestinal inflammation. No independent studies have investigated the difference in hBD2 expression between plasma cells, I/v-IEC and c-IEC. We speculate that the potential mechanism of hBD2 to prevent microbial attack might be related to the distance between cells and the intestinal cavity. The closer the cell is to the intestinal cavity, the higher the expression is. A study involving systemic administration via subcutaneous administration of hBD2 in the scapular region in mice found that recombinant hBD2 reduced inflammation, improved disease activity indices and prevented colitis-associated weight loss.^70^ And another study demonstrated a potential improvement in DSS-induced changes in paracellular permeability and mucosal lesions through the intrarectal administration of pBD2, which may impact the activation of NF-κB signaling.^69^ However, to date, there have been no studies of hBD2 in clinical trials in IBD. Given the differences in the expression of hBD2 in cases of UC, ileum CD, and colonic CD, these three clinical phenotypes may respond differently after hBD2 treatment. We speculate from our previous description that a protective effect of hBD2 therapy might be observed more often in UC or colonic CD than in ileal CD. Nonetheless, it appears that no related studies have been conducted thus far.

The expression of hBD1 is constant in the intestinal epithelium, and its expression levels remain unchanged in patients with IBD.^337^ Despite this, the precise function and mechanism of hBD1 concerning IBD have not been fully elucidated. hBD3 and hBD4 are like hBD2 and are noticeably increased in expression levels within the colon of patients with UC and CD.^326^ This observation may be because hBD2, hBD3 and hBD4 are inducible rather than constitutively expressed. However, in patients with IBD, the concentration of hBD3 and hBD4 are much lower than hBD2, and there is no significant difference in serum hBD3 and hBD4 (ref. ^337^). This suggests that hBD3 and hBD4 may be able to regulate local immunity. In addition, Meisch et al. investigated the distribution of hBD3 in the terminal ileum of healthy individuals and patients with CD. According to their findings, in the healthy small intestine, hBD3 is primarily observed in the luminal surface of the intestinal epithelium, as well as inside PC granules. However, in cases of CD, hBD3 relocates to the basolateral surface of the villus epithelium and accumulates in the lamina propria of the terminal ileum.^326^ We speculate that in patients with CD, hBD3 may, on the one hand, resist the microbial attack on the surface of the intestinal cavity and, on the other hand, enter the lamina propria and perform chemotaxis to recruit immune cells. Like with hBD2, there are still no clinical trials of hBD3 and hBD4 to treat IBD.

##### α-defensins: focusing on HD5

HD5 and HD6 are secreted mainly by PCs located in the small intestine and ileum, with a small amount coming from IECs.^338^ PCs continuously express HD5 and HD6 to protect nearby epithelial stem cells situated at the base of the crypts, thereby maintaining barrier integrity.^337^ Nevertheless, in IBD, the microbes and their metabolites and inflammatory factors interact to destroy PCs and IECs, thus disrupting HD5 and HD6 expression.^338,339^ Multiple studies have demonstrated a significant reduction in ileal HD5 and HD6 levels in patients with CD.^340,341^ As a result, antibacterial activity mediated by HD5 and HD6 is disrupted, resulting in a massive microbiome’s severe invasion of the intestinal mucosa and destruction of the epithelial barrier. Interestingly, patients with UC and colonic CD exhibit a significant increase in HD5 levels in their colon.^328,342–345^ This is mainly due to the absence of PCs in the colon of healthy people. However, after the occurrence of IBD, PC translocation hyperplasia occurs in the colonic crypts of patients with UC and colonic CD.^337,344,346^ We speculate that the possible mechanism is the colonic defensive response to microorganisms after the occurrence of IBD. Multiple mouse and cell studies have consistently confirmed the therapeutic effect of HD5 on colitis. For example, Shukla et al. found that HD5 administration improved ethanol- and colitis-triggered dysbiosis, inflammation response and barrier defects in the small intestine and colon.^347^ In addition, Zeng et al. created the recombinant NZ9000SHD-5 strain by transfecting the DEFA5 gene vector of pN8148-SHD-5 into *Lactococcus lactis* (*L. lactis*), which continuously produces mature HD5 (ref. ^348^). They found that NZ9000SHD-5 ameliorates intestinal damage and inflammation in mouse with DSS-induced colitis compared to the *L. lactis* + DSS group. These direct HD5 supplementation trials suggest that increased defensin expression is a potential avenue to treat colitis. Indeed, in a randomized clinical trial of anti-TNF therapy in patients with UC, HD5 was significantly upregulated in those who responded to the therapy compared to those that did not, with a lower microflora imbalance index in the responders.^349^ This suggests that the rise of HD5 may play a vital role in successfully treating UC with anti-TNF therapy. However, this needs to be confirmed experimentally; for example, in HD5 transgenic and knockout mice in a colitis model.

Unfortunately, to date, no clinical trials for IBD utilizing HD5 have been reported. However, some clinical retrospective and correlation studies have revealed the mechanism of PC regulation of HD5 and HD6 expression. This is helpful as it would then allow the targeting of the pathway of HD5 secretion by PCs as an additional means to treat IBD, to develop related inhibitors or agonists and to provide a solid foundation for the clinical application of HD5. For example, NOD2, a significant risk factor for ileal CD, is highly expressed in PCs, as shown by genome-wide association studies (GWASs).^104,350,351^ Economou and colleagues performed a meta-analysis and found that the CD risk is significantly increased in individuals with two mutated *Nod2* alleles (17.1-fold) and *Nod2* heterozygotes (2.4-fold).^352^ The mRNA expression of *DEF5A* (the gene encoding HD5) in PCs is significantly reduced in patients with a *Nod2* mutation compared to patients with CD expressing wild-type *Nod2* (ref. ^62^). These data suggest that NOD2 directly regulates HD5 in PCs to prevent CD and enhance mucosal protection. However, the *Nod2* mutation does not fully explain the downregulation of HD5. This is because healthy patients with *Nod2* mutations have higher HD5 expression levels than patients with CD expressing wild-type *Nod2* (ref. ^62^). In addition, the *DEFA5* gene promoter in PCs lacks NF-κB binding sites, indicating NOD2 is not directly involved in *DEFA5* gene transcription,^62,337^ suggesting that other factors also influence the regulation of HD5. Notably, Wnt signaling regulates the positioning, differentiation and maturation of PCs.^353^ Blocking the Wnt signaling pathway disrupts HD5 production in PCs and induces CD.^104^ This is because HD5 is a transcriptional target of TCF1 and TCF4, which act downstream in Wnt signaling, and thus is directly regulated by Wnt signaling in PCs.^104,271,354^ Both adult and child patients with CD exhibit a decrease in the expression of TCF1 and its active isoforms, confirming its role in CD pathology.^244,355^ Reduced expression of TCF4 is also associated with reduced expression of HD5 in PCs in patients with ileal CD irrespective of the degree of inflammation. Nevertheless, this association is not observed in patients with colonic CD or UC. Moreover, in *Tcf-4* knockout mice the α-defensins expression and bacterial killing activity were lower compared to wild-type mice, and in both species the reduced defensins expression occurred independently of the *NOD2* genotype.^340^

Similarly, HNP1-3 expression is also dysregulated in IBD. Multiple studies have repeatedly confirmed that patients with IBD highly express HNP1-3 and patients with UC have significantly higher expression than patients with CD.^340,356^ It is worth noting that experiments in mice have confirmed that HNP1 has dual effects. On the one hand, low doses of HNP1 (5 μg/day) can ameliorate DSS-induced colitis.^78^ On the other hand, high doses of HNP1 (100 μg/day) can promote a macrophage-driven inflammatory response and aggravate the progression of DSS-induced colitis.^357^ In addition, data from clinical samples showed that individuals with active UC have significantly higher expression of HNP1 compared to those with UC in remission. Kanmura et al. confirmed that an increased gene copy number of HNP1-3 and the severity of UC are positively correlated.^358^ These data suggest that HNP1-3 may be a risk gene for severe UC, and its high expression in patients with UC may induce a hyperinflammatory response. However, it is still challenging to know where the critical concentration of HNP1-3 is for the concentration-transition-dependent effect in patients with UC and whether to consider the concentration between HNP1-3 alone or the concentration of the three in total. These answers will require further studies in patients with UC in remission.

#### Defensins in diabetes and obesity

Type 2 diabetes is closely linked to obesity, which is expected to affect 1 billion people worldwide by 2030 (ref. ^359^). Evidence of dyshomeostasis of defensin in serum and tissues of patients with diabetes has been reported. For example, hBD1-3 is down-regulated, and HNP1-3 is upregulated, in the serum of individuals with type 1 diabetes (T1D).^360–363^ According to a prospective study examining cardiovascular risk factors, individuals belonging to the highest quartile for plasma HNP1-3 show a significant correlation with being leaner, more insulin sensitive and possessing lower levels of total and LDL-cholesterol.^364^ On the other hand, those belonging to the lowest quartile for circulating HNP1-3 lack these benefits.^364^ Moreover, even after considering the factors of age, BMI, insulin sensitivity and smoking, the links with serum lipids remain solid.^364^ Another investigation conducted by Liu et al. found that HNP1 inhibits hepatic gluconeogenesis via a c-Src-dependent pathway, resulting in lowering blood glucose concentration in normal mice and Zucker diabetic fatty rats.^71^ In addition, a low number of HNP1-3 gene copies may increase the risk for renal dysfunction,^83^ which is closely related to diabetes.^365^ These data suggest that HNP1-3 has a practical clinical significance in the control of blood lipid levels and treating diabetes-related diseases. Of note, various studies have pointed to the role of HD5 in both obesity and diabetes. For example, the levels of HD5 in the jejunum have been found to have an inverse correlation with obesity in humans.^366^ In addition, when mice are fed a high-fat diet and are deficient in vitamin D, there is a decrease in the expression of the murine analog, α-defensin-5. Functionally, mice with α-defensin-5 knockout experience more severe liver steatosis and metabolic disorders than the HFD-fed mice. However, when these mice were given exogenous HD5, observed symptoms improved, indicating that the protein is an essential regulator of metabolic balance.^367^ In addition, Larsen et al. fed mice a 60% HFD for 13 weeks and treated them with physiologically relevant levels of HD5 (0.001%) or vectors for 10 weeks. They found that HD5-treated mice show better glucoregulatory performance, as well as improved plasma and liver lipid levels in comparison to those treated with vectors.^72^ These findings demonstrate that the implementation of human defensins may hold promise in enhancing host metabolism, as well as mitigating the commonly related triad of dyslipidemia, obesity and diabetes. Moreover, clinical sample data and in vivo studies in mice and in vitro cell experiments also support the therapeutic benefits of defensins in treating obesity and diabetes. Nevertheless, no trials have been conducted in people with related diseases. The difficulty in producing defensins remains a significant challenge. However, the Wehkamp laboratory recently demonstrated that intestinal proteases digest HD5 to form peptide fragments with potential antimicrobial activity.^107^ This newly generated peptide fragment may replace full-length peptides, providing a solution for the clinical use of HD5 active fragments.

#### Chronic inflammatory lung disease and defensins

The lungs inspire numerous pathogens daily. As defensins play a vital role in the fight against pathogens and mediate immune response, the role of specific defensins in regulating inflammatory lung disease has been investigated. Multiple studies have confirmed that single-nucleotide polymorphisms and copy number variations of DEFB1 and DEFB2 are associated with chronic obstructive pulmonary disease (COPD) and asthma.^368–373^ The ile38 variant (untranslated regions) of hBD1 was detected in 15.0% of patients, while only 2.8% of healthy individuals carried this variant. Its presence has been found to be significantly associated with the disease.^369^ Furthermore, over 80% of patients with this hBD1 ile38 variant reported experiencing sputum production for more than three months during their follow-up period. This suggests that the ile38 variant of hBD1 exacerbates the disease state of COPD. In addition, Andresen et al. and Baines et al. reported that hBD1 expression is elevated in bronchia biopsies of patients suffering from asthma or COPD.^372,374^ This rise in hBD1 expression is associated with COPD’s pathological changes and disease severity.^372,374^ Similar studies were replicated with hBD2. For example, levels of hBD2 were observed to correlate with IL-8 level as well as COPD severity.^375^ This result implies that it is an effector in the innate immune response involved in COPD’s pathogenesis. However, studies have also reported that hBD2 is decreased in central airways of COPD individuals who smoke, but not in distal ones.^376^ In addition, the concentration of hBD2 in pharyngeal washing fluid and sputum of smokers or former smokers is markedly lower than individuals who never smoked.^377^ Upon co-infection with viruses and bacteria, individuals with COPD have shown a decrease in the production of hBD2. Administering recombinant hBD2 has proven to be effective in reducing lung neutrophilia caused by exposure to cigarette smoke, while still maintaining proper immune function and promoting an appropriate response to bacterial stimuli.^378^ In addition, oral treatment with hBD2 is beneficial in mitigating the effects of house dust mite challenge in a murine asthma model, whether administered prophylactically or therapeutically.^373,379^ We speculate that the upregulation of hBD2 in COPD will play a pro-inflammatory role in inducing lung cell death. Due to the impaired immune function in patients with COPD, when smoking or when there is a large challenge by bacteria and viruses, hBD2 already expressed in COPD will be neutralized. In such cases apoptotic epithelial cells will not be able to continue to express hBD2. Thus, the immunomodulatory, antibacterial and antiviral effects of hBD2 are inhibited, and the inflammatory response of COPD is further aggravated.

#### Periodontitis and defensins

Periodontitis, which is responsible for a large percentage of tooth loss among adults, affects approximately 47% of adults.^380^ Defensins are biomarkers for the early diagnosis of periodontitis and regulate the interaction between the subgingival microbiota and host tissues.^381^ Research has indicated that the concentration of both α-and β-defensins in the saliva of individuals with chronic periodontitis is higher than in healthy cases.^382–387^ In addition, a recent bioinformatics study predicted that hBD1 might be able to bind effectively to the virulence factors of red complex bacteria in periodontitis, potentially reducing the severity of the infection.^388^ In vivo, hBD3 inhibits the severity of periodontitis induced by *Porphyromonas gingivalis* in mice and decreases osteoclast formation, while less alveolar bone loss was also observed.^387^

#### Cancer and defensins

The role of defensins in cancer development and progression has been a topic of intensive research, with some noteworthy findings.^389,390^ Human tumor tissue clinical samples show remarkable changes in the expression of defensins, while in vivo studies in mice and in vitro studies of related cancer cells show that defensins have anticancer and tumor progression effects. For example, in one study 82% of prostate cancer clinical tissues showed complete loss or minimal expression of hBD1 protein, while adjacent benign epithelial cells expressed it normally.^297^ Similarly, 90% of clinical renal cell carcinoma tissues show cancer-specific deletion of hBD1 protein.^297^ In addition, clinical samples of renal and prostate cancer reveal the discovery of three novel hBD1 promoter mutations.^298^ Synthetic hBD1 and overexpression of hBD1 can promote the death of bladder cancer cell and the renal cancer cell.^298^ These data suggest that hBD1 could possibly function as a tumor suppressor in urological cancers. In addition, hBD1 inhibits tumor growth of oral squamous cell carcinoma (OSCC) and lung cancer in vitro and in vivo in mice. However, hBD1 production appears to be closely linked to cancer type. For example, hBD1 expression is reduced in prostate, kidney and skin basal cell carcinoma (SBCC) and skin squamous cell carcinoma (SSCC), colon, liver, and OSCC but upregulated in lung squamous cell carcinoma (LSCC) and adenocarcinoma (AC). This pattern is further supported by studies indicating that serum hBD1 levels are notably elevated in patients with lung cancer as opposed to healthy people and patients with pneumonia.^297,298,391–395^

It should be noted that further confirmation of the potential therapeutic benefits of hBD1 is still lacking in transgenic animal studies and in vivo studies in primates. Phan and colleagues found that the sequence of hBD3 possesses a homologous β2-β3 loop that binds phosphoinositides to promote cytolysis of tumor cells.^396^ Continuous infusion of hBD3 in mice shows a remarkable inhibition in tumor growth in Lewis lung carcinoma cells and inhibits migration of colon cancer cells.^397,398^

However, some paradoxical results of hBD3 promoting tumor progression have also been found. For example, hBD3 contributes to the carcinogenesis of cervical cancer, HNSCC and OSCC via the activation of NF-κB signaling.^399–403^ Notably, defensins may also be regulated by bacteria or viruses before indirectly influencing cancer development. For example, *Porphyromonas gingivalis*, associated with oral cancer progression, actively triggers the transcription of α-defensins in oral tumor cells, which in turn is thought to promote the proliferation of these cells.^389^ In contrast, HD5 and HD6 are protective against colon cancer.^404–406^ For example, HD5 expression is reduced in colon cancer tissues from patients, and prognostic results indicate that patients with high HD5 expression have significantly longer survival than patients with low HD5 expression.^405^ HD5 overexpression also inhibits tumor growth in nude mice. Similarly, HD5 also inhibits the growth of gastric cancer.^407^ We summarized the expression of defensins in different cancer types in Fig. 6b.

Overall, the immunomodulatory activity of defensins offers the potential for them to be an effective anticancer therapy. Nonetheless, the development of defensin-based cancer therapies is complicated by the conflicting roles of defensins in different cancers. Future research is required to identify unique active structures of defensins that can be used to develop derived peptides with the discriminatory ability to target specific cancers.

### Clinical trials of defensins

#### Brilacidin

Brilacidin, a synthetic defensin mimetic obtained from plants, has undergone extensive clinical testing involving more than 500 human patients for the treatment of various conditions, such as acute bacterial skin and skin structure infection (ABSSSI), UC, COVID-19 and oral mucositis (OM). For example, in vitro testing has revealed that brilacidin exhibits broad-spectrum antiviral activity, particularly against multiple human coronaviruses, including SARS-CoV-2. However, it does not possess antiviral activity against influenza or enterovirus.^408–410^ According to previous research, brilacidin has a dual anti-SARS-CoV-2 mechanism of action that involves targeting host cell surface heparan sulfate proteoglycans to prevent viral attachment and to inactivate viral particles.^408^ In fact, the US FDA has granted Fast Track status for brilacidin for COVID-19 treatment and a Phase 2 clinical trial (NCT04784897) on hospitalized patients has been conducted.^411^ Although the study did not meet its primary endpoint, the recovery time was significantly reduced for patients who received study treatment less than seven days after showing symptoms of COVID-19. Regarding two secondary endpoints, a higher number of patients treated with brilacidin (5-dose group) experienced clinical improvement by ten days after treatment initiation, as assessed using the National Emergency Warning Score 2 (NEWS2) criteria. The mean change in NEWS2 baseline was more remarkable for the brilacidin-treatment groups at all evaluated time points.

Brilacidin also effectively prevents and controls OM in patients undergoing head and neck cancer (HNC) chemotherapy. A Phase II clinical trial of brilacidin for this circumstance (NCT02324335) showed that patients with HNC who self-administered brilacidin three times a day for 7 weeks significantly reduced the incidence of OM compared with placebo (from 60 to 36.8%).^412^ Two randomized, phase II trials (NCT02052388 and NCT01211470) indicate that a single dose of intravenous brilacidin is just as safe and effective as FDA-approved daptomycin for the treatment of ABSSSI, with an early clinical response (7-day) rate of 90% (refs. ^413–415^).

Aside from its use in COVID-19, ABSSSI and OM, brilacidin is currently being developed as a preventative measure for UC. Most patients with UC that were treated with brilacidin achieved induction of clinical remission.^416^ After administering brilacidin, there were no reports of Serious Adverse Events (SAEs) and it was generally well-tolerated.^416^ In animal models, brilacidin also demonstrated a potential therapeutic effect in treating keratitis (topical drops) and pulmonary infection (intraperitoneal injection) induced by *Aspergillus fumigatus*.^417,418^ These studies all show the clinical potential of brilacidin, although further investigation is needed.

#### Pezadeftide (HXP124)

HXP124 is a novel plant defensin being clinically developed by Hexima Ltd as a novel topical candidate for treating onychomycosis.^419,420^ To evaluate its efficacy, a Phase I/IIa clinical trial was conducted using pezadeftide (Australian Clinical Trials ID: ACTRN12618000131257) in a double-blinded, randomized study with multiple ascending doses.^420,421^ Patients who received daily topical application of pezadeftide for 6 weeks were found to have reduced infection area compared to those receiving current best-in-class therapies, with a shorter treatment time and excellent safety profile.^420,421^ The clinical data from this trial demonstrated a 69% Mycological Cure rate at 12 weeks, a vast improvement over the 29% rate achieved by the control group.^420,421^ These results show very promising clinical efficacy. Hexima has since raised $11 million and initiated Phase IIb testing of 2% pezadeftide in three active arms to determine the optimal dosing frequency and evaluate further its safety and efficacy.^422^

## Hurdles to the development of defensins-based therapy

From the extensive evidence presented previously, the role of defensins and targeted therapies offer new hope, but most natural defensins may not be suitable as drugs for direct application. The main challenges lie in the following areas. (1) The effect of defensins in vivo requires a suitable local microenvironment. The direct antibacterial activity requires high local concentrations, and many defensins exhibit specific cytotoxicity and inflammatory responses at high concentrations,^17^ as described in the Regulation of Cell Death section. In addition, specific disease environments lead to significant changes in mucosal pH and salt ion concentrations that do not allow defensins to function.^423–426^ (2) Natural defensins are sensitive to protease-mediated inactivation. The human body contains nearly 600 proteases^427^ that, depending on the structure of the defensin, work together to exert proteolytic activity. The stability of peptidases can be significantly improved through various methods, such as altering specific amino acid residues or modifying the peptide skeleton through techniques like protection or cyclization of the amino and carboxyl terminus.^428^ Another approach is the utilization of protease inhibitors. (3) The formulation and delivery of defensin drugs have not been fully realized. Most current HDP-based therapies are applied externally; for example, to treat skin and respiratory diseases.^17,429,430^ This is because current defensin drugs lack appropriate drug-like properties. Without the participation of the formulation and delivery system, the absorption efficiency, delivery efficiency and metabolic cycle of defensin drugs will be directly affected. Therefore, for defensin drugs to be delivered to the body (by oral or injection), a way must be found to overcome these barriers. One way to address this issue is to consider alternative delivery methods such as liposomes, polymer nanoparticles, carbon nanotubes and similar materials. For example, Krishnakumari et al. synthesized the N-terminal myridamylated Phd1-3 peptide, MPhd1-3. They found that it was more active against *S. aureus* and remained active in the presence of 150 mM NaCl, whereas hBD1-3 was not.^431^ In addition, Lei et al. designed a nanobiotic component assembled from C-terminally myristoylated HD5 (HD5-myr).^432^ In vitro and in vivo experiments have revealed that HD5-myr has an extraordinary efficacy in disrupting the structure of bacterial membranes or cell walls, and its antibacterial activity is considerably higher in the presence of sodium chloride or serum when compared to HD5 (ref. ^432^). According to Yuan et al., a three-dimensional porous structure was introduced onto polyether ether ketone through sulfonation, which was then coated with mBD14 to create a long-lasting antimicrobial coating (SP-mBD). The newly formed coating showed a high efficiency in eradicating a wide range of bacteria while promoting osseointegration.^68^ (4) The high cost of synthesis or expression. Currently, most of the clinical and experimental applications of defensins are based on chemical synthesis methods, including solid-phase peptide synthesis (SPPS) and liquid-phase peptide synthesis (LPPS).^425^ Of these techniques, SPPS is the most widely employed. However, SPPS has some technical, cost and environmental challenges. For example, SPPS has difficulty synthesizing defensins containing many hydrophobic side-chain amino acids. This is because the structure of defensins contains many β folds or α helices, which have high hydrophobicity, resulting in high aggregation in water-based solvents, thus producing low solubility intermediates that affect the subsequent purification process.^433,434^ It is worth noting that with the development of defensins fragmentation research, some small fragments of defensins, such as HD5_1-9_ (refs. ^107,435^) and HNP4_1-11_ (ref. ^154^) have been shown to replace full-length defensins in antibacterial activity. We think this could be a pivotal way to overcome the limitations of high synthesis costs and that small fragments are more helpful in finding suitable delivery materials in vivo.

## Nutritional regulation strategy

Due to defensins being toxic to mammalian cell membranes at high concentrations, the transcription, translation, and activity of most defensins are strictly controlled to avoid excessive immune responses. Given these mechanisms, developing strategies to regulate the expression and secretion of defensins would be significant. Here, we review interventional methods for handling endogenous defensins, including fatty acids, amino acids, microelements, plant extracts and probiotics (Table 4).

**Table 4:** Regulation effects of nutrients on the expression of defensins

	Nutrient	Object	Defensins	Pathway	Reference
Fatty acid	SCFAs	HT29	hBD1-2		^439^
	SCFAs	Mice IEC	mBD1/3/4	GPR43-mTOR/STAT3	^442^
	Sodium phenylbutyrate	IPEC-J2	pBD1-3	TLR2/4-NF-κB	^443,444^
	Caprylic acid and nonanoic acid	IPEC-J2	pBD1-2	Acetylation H3K9	^441^
	Sodium phenylbutyrate	IPEC-J2	pBD1-3	HDAC inhibition	^443^
	Butyrate	Macrophage and intestine	pBD2-3	HDAC inhibition	^446^
Amino acid	Leucine	Mice PCs	Cryptdin1		^453^
	l-isoleucine	IPEC-J2	pBD1-3	Sirt1-ERK-90RSK	^455^
	l-isoleucine	Caco-	hBD2		^456^
	l-arginine	Intestine and oral	pBD2-3		^457^
	Isoleucine	Caco-2	hBD2	GPCR-ERK	^456^
	l-isoleucine	Bovine kidney EC	β-defensins	NF-κB/rel	^458^
Microelements	25OH vitamin D3 (25D3)	keratinocytes	hBD4	TLR2-NF-κB-Cyp271b/Cyp24-VDR	^459,460^
	1,25OH vitamin D3	IEC and monocyte	AvBD3/6/9	VDR	^461^
	Zn^2+^	IPEC-J2	pBD1-3		^454^
Plant extracts	ITF	Intestine	mBD1		^467^
	Avocado sugar	keratinocyte	hBD2	TLR2-ERK/MAPK	^468^
	EGCG	IPEC-J2	pBD2	ERK1/2/p38-MAPK	^464^
	Dehydroandrographolid	HCT-116	hBD2	p38-MAPK	^470^
	Reishi	Intestine	RD5-6	TLR4	^471^
	β-Glucan	Ovine ruminal ECs	SBD1	Dectin-1-Syk-NF-κB	^469^
	Black tea extract and theaflavins	Oral ECs	hBD1/2/4		^466^
Probiotics	*Lactobacillus*	Caco-2	hBD2	NF-κB and AP-1	^473^
	*Escherichia coli* Nissle 1917	Fecal	hBD2	NF-κB and AP-1	^476–478^
	*Bacillus subtilis* yb-1114246	Intestine	AvBD1	TLR2-NF-κB	^435^
	*Clostridium butyricum*	IPEC-J2 and Intestine	pBD1-3	TLR2	^479^
	*Lactobacillus casei strain Shirot*	Caco-2	hBD2		^504^
	*Bacillus subtilis BYS2*	Intestine	AvBD1/6		^505^
	*Lactobacillus salivarius B1*	Intestine	pBD2		^506^
	*Lactobacillus rhamnosus MLGA*	Intestine	AvBD9		^507^
	*Bifidobacterium longum spp. S12*	Intestine and cancer cells	hBD2		^508^
	*Lactobacillus helveticus SBT2171*	Caco-2	hBD2	TLR2-JNK	^472^

### Fatty acids

**Fig. 7:**
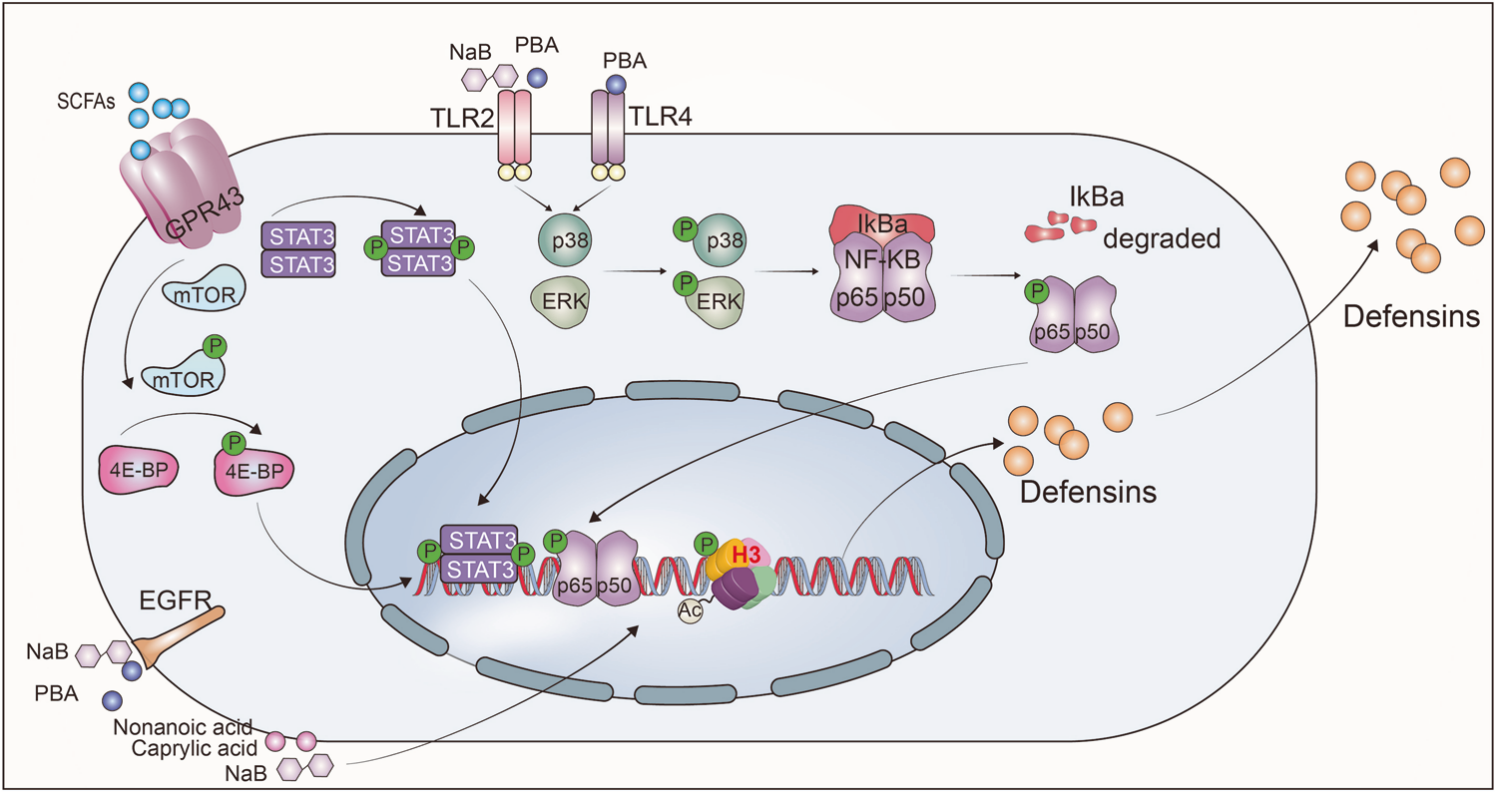
Signaling pathways of fatty acids-regulated expression of defensins. SCFAs induce the expression of β-defensins via GPR43-STAT3 and GPR43-mTOR-4E-BP signals; sodium butyrate (NaB) induces the expression of β-defensins via TLR2-p38/ERK- NF-κB, HDAC inhibition and EGFR signals; sodium phenylbutyrate (PBA) induces the expression of β-defensins via TLR2/TLR4-p38/ERK-NF-κB and EGFR signals; Caprylic acid and nonanoic acid induce the expression of β-defensins via HDAC inhibition-acetylation H3K9

Fatty acids (FAs), the main components of lipids, undergo various metabolic processes after absorption in the gut. Proper lipid metabolism in the gut is essential to ensure adequate energy for the body’s organs. In humans, defects in the absorption of lipids can cause severe symptoms, including IBD and IBS.^436,437^ The length of the aliphatic hydrocarbon chain of free FAs has been found to have a negative correlation with the ability of FAs to induce defensin expression.^438^ Undigested dietary fiber in the large intestine undergoes bacterial fermentation, yielding short-chain fatty acids (SCFAs), such as acetate, propionate, and butyrate. They enhance the expression of hBD1 and hBD2 in IECs^439^ (Fig. 7). In pigs, caprylic acid and nonanoic acid, both medium-chain fatty acids, significantly increase pBD2-3 and pBD1-2 expression, respectively, in porcine intestinal enterocytes (IPEC-J2) and decrease bacterial translocation, with augmented antibacterial activity^440,441^ (Fig. 7).

The expression of defensins mediated by SCFAs involves multiple signaling pathways. For instance, GPR43 mediates SCFA-regulated β-defensins-1, 3, and 4 expressions in IECs via activating mTOR and STAT3 (ref. ^442^) (Fig. 7). Sodium phenylbutyrate, an aromatic SCFA, promotes the expression of pBD1-3 through the TLR2/4-mediated NF-κB pathway^443,444^ (Fig. 7). In addition to TLRs or GPCRs, histone modification is involved in FA-induced expression of defensins. For example, caprylic and nonanoic acid attenuate histone deacetylase (HDAC) activity, leading to an elevation in the acetylation level of H3K9 and an upregulation of pBD1 and pBD2 (ref. ^441^) (Fig. 7). Sodium phenylbutyrate and butyrate further amplify defensin secretion through histone deacetylation and STATs phosphorylation in IPEC-J2 cells and crypt organoids^443,445^ (Fig. 7). In addition, in our previous study we found that butyrate upregulates pBD2 and pBD3 to enhance disease resistance, including promoting the removal of harmful bacteria and improving inflammation caused by *E. coli* O157:H7 infection in piglets via HDAC inhibition^446^ (Fig. 7). These results indicate that FAs are adequate to induce the expression of defensins via multiple signaling pathways, in which histone acetylation may be the target of FAs to activate these signaling.

### Amino acids and microelements

**Fig. 8:**
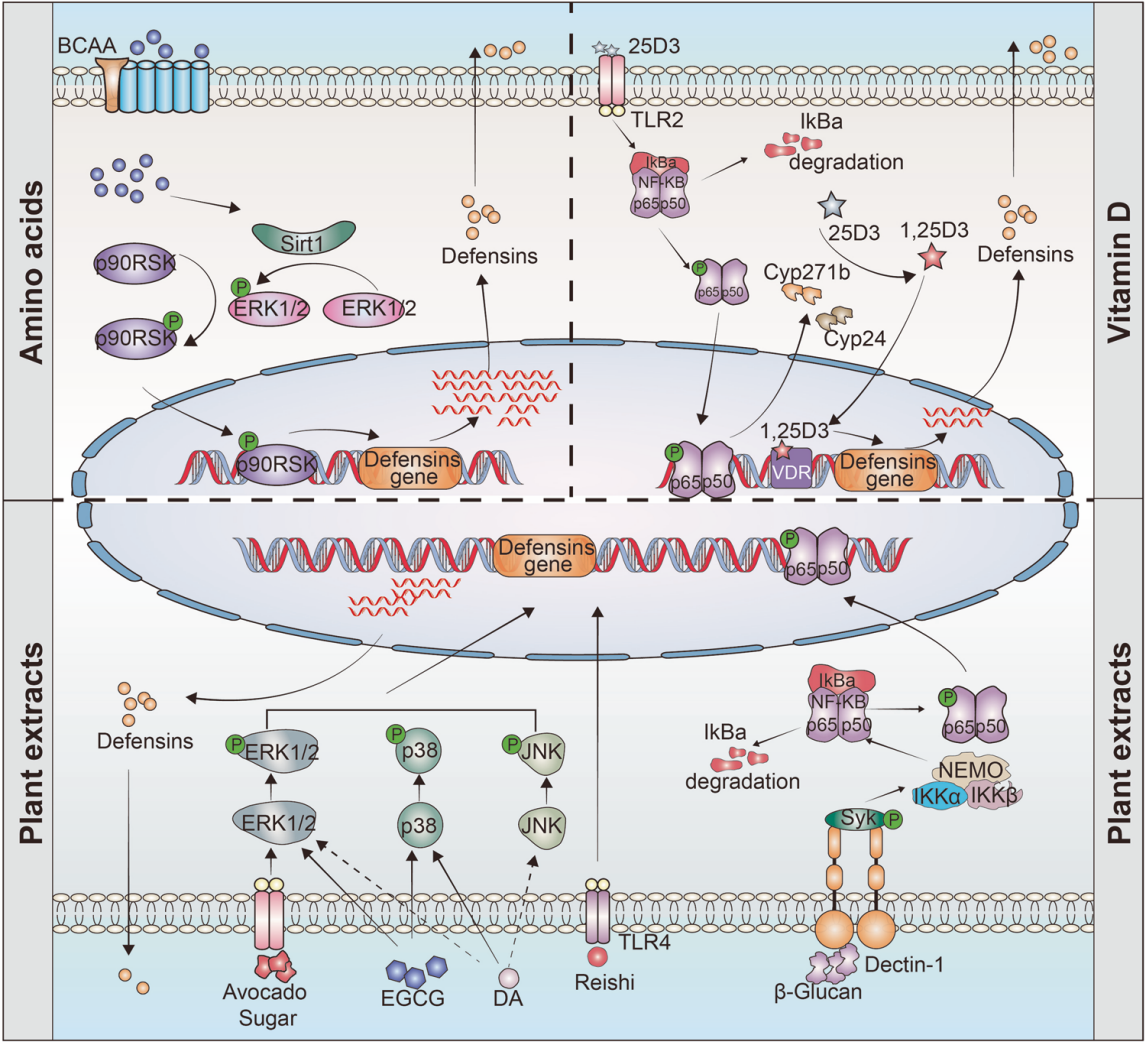
Signaling pathways of amino acids, vitamin D and plant extracts regulate the expression of defensins. Top left**:** l-isoleucine induces the expression of β-defensins via the SIRT1/ERK/90RSK signals, and G-protein coupled receptor-ERK pathways. Top right**:** 25OH vitamin D3 (25D3) induces the expression of β-defensins via TLR2-NF-κB-CYP271B/CYP24-VDR signals. Bottom**:** Avocado sugar via TLR2-ERK1/2, EGCG, and DA via p38, Reishi via TLR4, and b-Glucan via Dectin-1-Syk-Ikk-NF-κB regulate the expression and section of defensins

Amino acids are critical regulators in many metabolic processes. Amino acid transport in the intestine is crucial to supply sufficient amino acids to all tissues and to maintain the homeostasis of plasma amino acid levels.^447^ It has been previously shown that defensins play an essential role in the IBD-induced disorder of amino acid metabolic profile in blood, feces and the intestine.^448–451^ Consistent with these results, some amino acids promote intestinal barrier function and intestinal endocrine homeostasis via a defensins-related mechanism.^452,453^ Takakuwa et al. found that leucine administration significantly induces α-defensins secretion from the PCs of the small intestine, compared with phosphate-buffered saline and 19 other amino acids, in a dose-independent manner.^453^ Moreover, l-isoleucine and branched-chain amino acids (BCAA) administration enhances pBD1-3 levels in the small intestine and epithelial cells,^454,455^ as well as in human colonic epithelial cells.^456^ l-arginine administration promotes the expression of pBD2 and pBD3 in the ileum of weaned pigs.^457^ Interestingly, the ability of amino acids to induce defensins expression was related to not only the type of amino acid but also its isomer. For example, l-isoleucine could induce a 12-fold expression of β-defensins at a low concentration of 3 μg/mL, whereas d-isoleucine required a concentration of 200 μg/mL.^458^ Mechanistically, the MAPK pathway is related to the amino acid-induced expression of defensins. For instance, the expression of β-defensins induced by l-isoleucine is via the SIRT1/ERK/90RSK signals and GPCRs-ERK pathways in IPEC-J2 cells^455,456^ (Fig. 8). 25OH vitamin D_3_ (25D3) activates TLR2, which promotes the expression of CYP27b1 and CYP24, which, in turn, convert 25D3 into active 1,25(OH)_2_ vitamin D_3_, a ligand for the vitamin D receptor (VDR), ultimately leading to defensins transcription and therefore mediating an antimicrobial response^459,460^ (Fig. 8). Moreover, 25D3 can induce AvBD secretion in the avian embryonic gut.^461^ Like amino acids, zinc remarkably increases the mRNA and protein levels of pBD1-3 in IPEC-J2 cells.^454^ Nevertheless, the mechanism still needs further investigation.

### Plant extracts

Plant extracts are bioactive compounds extracted from plants with one or more biological functions, such as antimicrobial (bacteria, protozoa, and fungi), immunity and antioxidant activities.^462,463^ For example, green tea and vegetables are rich in epigallocatechin-3-gallate (EGCG), which helps to prevent the breakdown of recombinant hBD1 and hBD2. At the same time, it encourages their secretion in epithelial cells through the activation of ERK1/2 and p38-MAPK signal pathways^464^ (Fig. 8). Peony, a plant that is commonly used in traditional Chinese medicine, contains paeoniflorin (PF), a compound that increases the expression of hBD2 in bronchial epithelial cells to strengthen epithelial antimicrobial barriers. Mechanistically, this effect is achieved by upregulating the p38-MAPK, ERK, and NF-κB signaling pathways.^465^ Similarly, black tea extract and theaflavins attenuate IL-8 secretion and induce hBD1, hBD2, and hBD4 secretion in epithelial cells.^466^ In addition, fiber and carbohydrates, rich in plants, can regulate defensins expression and improve immunity. For example, Chen et al. found that supplementing with inulin-type fructan fibers (ITF) can lead to an increase in the expression of mBD1, which in turn may contribute to protection against autoimmune diabetes by regulating cytokine production and improving the ratio of Treg/Th17 (ref. ^467^). Avocado sugar modulates the hBD2 and hBD3 expression in human keratinocytes through TLR2 and ERK/MAPK activation^468^ (Fig. 8). β-Glucan from *Saccharomyces cerevisiae* activates the Dectin-1-*Syk*-NF-κB pathway to induce β-defensin-1 expression in the ruminal epithelial cells of sheep^469^ (Fig. 8).

Furthermore, plant-derived Chinese herbal medicines dehydroandrographolide (DA) and reishi are also effective regulators of defensin expression. Xiong et al. found that DA enhances innate intestinal tract immunity by increasing hBD2 expression in HCT-116 intestinal cells through the p38-MAPK pathway^470^ (Fig. 8). Reishi, a polypore fungus, enhances IgA secretion and the expression of RD5 and RD6 in the rat intestine via a TLR4-dependent signaling in a concentration-dependent manner (Fig. 8); however, it does not activate TNF-α. Therefore, supplementation with reishi may be a potential therapy to ameliorate intestinal infection.^471^

### Probiotics

Probiotics have shown potential as a therapy for gut inflammation, but their interaction with host defense defensins remains relatively unexplored. For instance, the S layer protein derived from *Lactobacillus Swiss* SBT2171 promotes the expression of hBD2 via the TLR2-JNK signal, thus providing a protective shield against infection.^472^ Other strains of *Lactobacillus* and probiotic cocktails like VSL#3 stimulate hBD2 secretion through the NF-κB and AP-1 signal, helping to reinforce intestinal barrier functions.^473^ Similarly, the probiotic *E. coli* Nissle 1917 boosts hBD2 expression via flagellin-mediated NF-κB and AP-1 pathways, enhancing the mucosal barrier against luminal bacteria.^474,475^ With beneficial outcomes observed in human clinical studies, *E. coli* Nissle 1917 appears to show promise.^476–478^

Selenium-enriched *Bacillus subtilis* yb-1114246 activates the TLR2-NF-κB signal to control intestinal β-defensins expression, thereby improving the immune status of the intestine.^435^ Our prior research indicates that *C. butyricum* binds to the adhesion sites of IECs, prompting the secretion of pBD1-3 by IECs.^479^ *C. butyricum* and pBD1-3 synergistically positively regulate the composition of intestinal microbiota and SCFA production, culminating in an improvement in intestinal immune function in weaned piglets.^479^ Over- or under-production of defensins can adversely impact intestinal integrity. However, the beneficial effects of probiotics in adjusting abnormal defensin levels, be it an increase or decrease, have been fairly consistent in aiding host recovery. A deeper understanding of the interactions between probiotics and defensins is necessary. This will facilitate the comprehensive analysis of dysregulation of defensin homeostasis and microbial crosstalk in various gastrointestinal diseases, which is vital for treating gastrointestinal diseases.

Although several nutrients have been shown to regulate the expression of defensins, this screening approach excessively relies on reproducible experiments. A high throughput screening method was recently developed, in which the capacity of up to 584 compounds to induce the expression of specific defensins, such as LL-37 and AvBD9, could be determined in one in vitro experiment.^480,481^ Such a screening approach will significantly accelerate the speed of discovery of nutrient-induced defensin expression.

## Conclusions

As a common defense mechanism among mammals, host-derived defensins comprise a critical innate immune barrier to external insults. A better understanding of the expression site, chemotactic activity, inflammation regulation, damage regulation and secretion regulation of host-derived defensins is critical to comprehending host defense mechanisms and disease processes. Although there is still a lack of solid clinical trials that adequately utilize the immune effectors of defensins in various diseases, both clinical and preclinical data obtained using mouse models highlight the vital role that defensins play in regulating the immune response. Meanwhile, in the future, the field should focus on exploring defensin functions and mechanisms in ameliorating specific diseases by establishing defensin knockout animal models or utilizing clinical samples. Moreover, as multiple defensins are present in the host, better tools and proteomic methodologies must explore how the synergies between defensins improve innate immunity or enhance resistance to infection. But even so, the combined data generated to date in the field point to a bright future for a role of defensins or their derivatives in the treatment of various human diseases.
